# Supplementary material for: A graph-based algorithm for RNA-seq data normalization
Source: PLoS One. 2020 Jan 24;15(1):e0227760. doi: 10.1371/journal.pone.0227760 (PMC6980396; doi:10.1371/journal.pone.0227760)
Supplement: S1 Supporting Methods — (PDF) [file pone.0227760.s001.pdf]

A graph-based normalization method for RNA-seq data
Supporting Methods

Diem-Trang Tran, Aditya Bhaskara, Balagurunathan Kuberan, Matthew Might

1 Characterization of validation data sets and simulated data set

In this work we presented a novel construction of validation data sets from real RNA-seq data, trying to preserve both biological and artifactual correlations that are critical for reference identification. The procedure (as described in the main text) starts with compiling a list of reference genes and differential genes. Reference genes are composed of ERCC spike-ins while differential genes are composed of signaling transduction pathway genes, minus the few of known house-keeping genes among them. Genes within a biological pathway are usually regulated together, resulting in co-expression. When included in the validation set, they provide some biological correlations that contest with the artifactual ones arising during the experimental process.

In constrast, simulation is the mainstream approach to evaluate RNA-seq normalization methods [1, 2]. This approach provides the platform to evaluate normalization methods in highly-specific application (differential expression analysis) and setting (exactly two conditions). To generate a simulated data set, we used the procedure and code from Evans et al. [2] to sample read counts from negative binomial distributions, with fold-change (k) set to 2, and the number of genes, number of samples, and percentage of differential genes set to those of the validation data set.

To characterize the data sets resulted from two different approaches, we surveyed the distribution of read counts and the patterns of correlations within reference genes, and in the whole data set. Regarding the distribution of read counts, most genes in the real data set are not expressed (or not high enough to be detected). In the simulated data set, most genes are detected at the medium level. Distribution of read counts in the mini validation sets has two major peaks, at zero and at the medium level (Supp Fig. S1). The correlation patterns are distinct between the validation set and the simulated data. Among the references, correlations in the validation sets are highly positive, while those in the simulated set are peaked around 0.5. There exist multiple clusters, including that of highly-correlated references in the validation set, while there are only two groups in the simulated counterpart.

2 Parametric choices

To reduce the computational demand of correlation calculations and downstream community detection, we applied a couple of filters to reduce the number of vertices and edges in the graph. First, only genes with positive read count in all of the samples were considered. This simple cutoff left out 85% of the genes, resulting in a much more compact set of vertices (Supp Fig. S2). Second, lowly expressed features were removed, due to low reliability of RNA-seq read-out at this levels. In addition to trimming more vertices, this step also helps reducing the amount of noise correlation (Supp Fig. S3), although the specific procedure is not critical. Third, edges were formed when correlation is greater than the threshold $t = 0.75$. Based on the distribution of correlations in

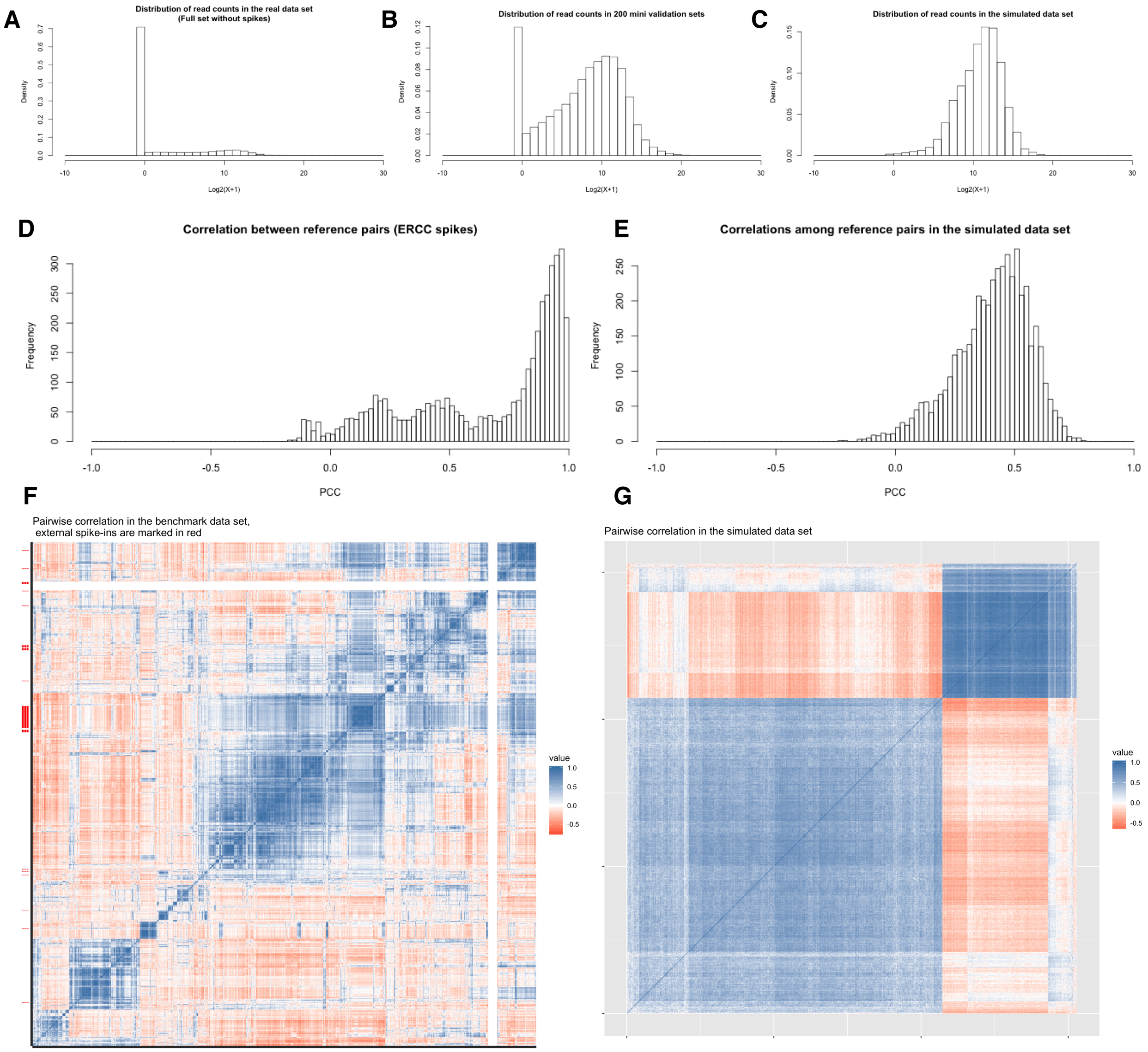

Supp Fig. S1: Characterization of validation data sets. (**A, B, C**) Distribution of read counts (log-transformed on read count added with a pseudo-count of 1) in real data set (Full set without ERCC spike-ins), validation data sets and simulated data set of equivalent size. (**D, E**) Pair-wise correlations among the experimental references (ERCC spikes) are highly positive while those among the simulated references are rarely beyond 0.75. (**F, G**) Complex groups of correlated genes are present in the benchmark data set but not in the simulated dataset.

the experimental and simulated data (Supp Fig. S1D-E), this threshold can reasonably distinguish meaningful correlations from the noise.

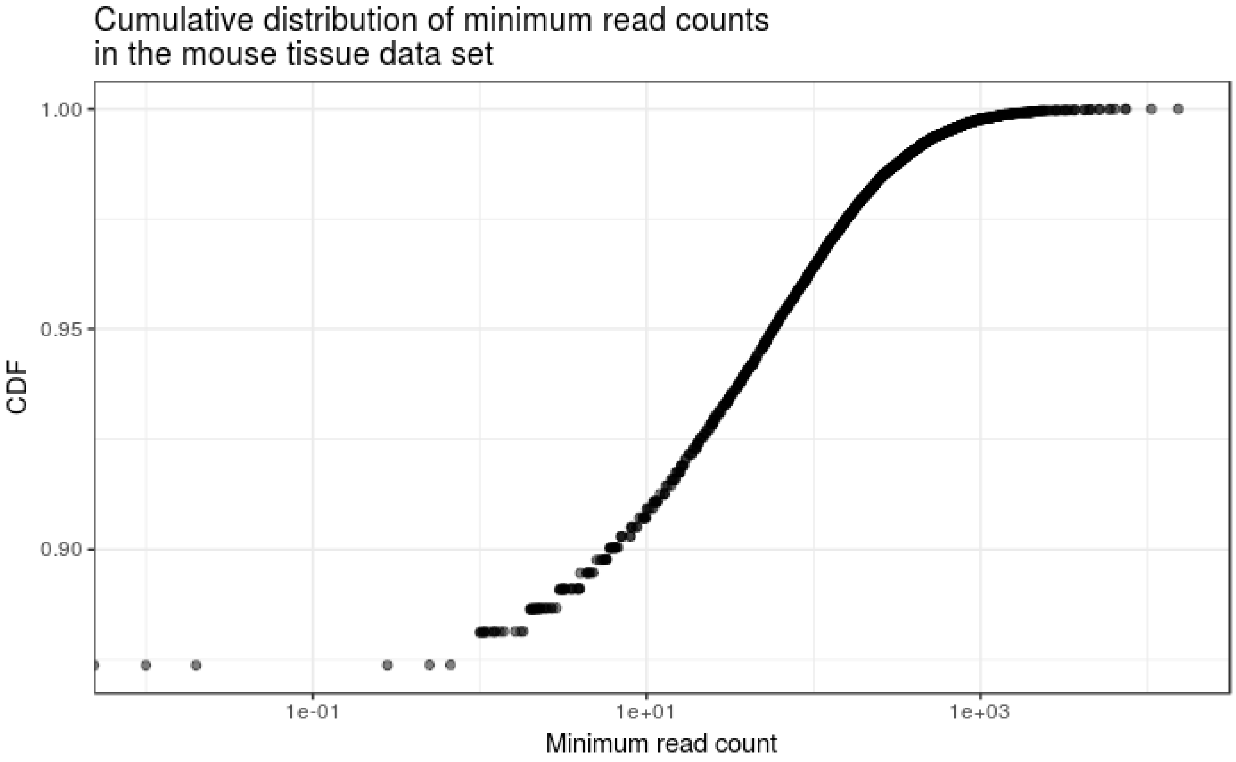

Supp Fig. S2: Cumulative distribution of minimum read counts in the *Full* data set

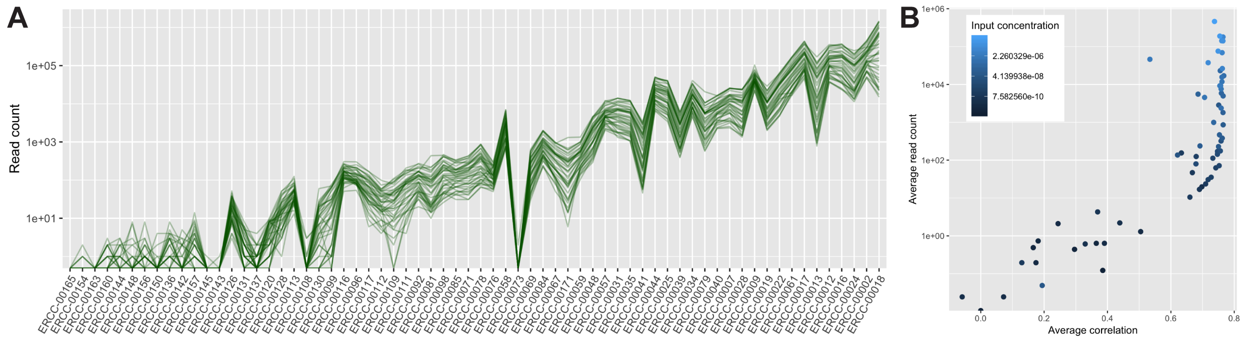

Supp Fig. S3: Pairwise correlations of all 96 RNA sequences in the ERCC spike-ins set. (**A**) Spike-ins are ordered on the x-axis in increasing input concentration from left to right. Spike-ins added at very low concentration could not be detected reliably, resulting in noisy correlation. (**B**) When spike RNAs can be detected with sufficient reads, the average correlation (with all others) is consistently high.

References

[1] Dillies MA, Rau A, Aubert J, Hennequet-Antier C, Jeanmougin M, Servant N, et al. A Comprehensive Evaluation of Normalization Methods for Illumina High-Throughput RNA Sequencing Data Analysis. Briefings in Bioinformatics. 2013;14(6):671–683. doi:10.1093/bib/bbs046.

[2] Evans C, Hardin J, Stoebel DM. Selecting Between-Sample RNA-Seq Normalization Methods from the Perspective of Their Assumptions. Briefings in Bioinformatics. 2017;doi:10.1093/bib/bbx008.
