# Supplementary material for: A graph-based algorithm for RNA-seq data normalization
Source: PLoS One. 2020 Jan 24;15(1):e0227760. doi: 10.1371/journal.pone.0227760 (PMC6980396; doi:10.1371/journal.pone.0227760)
Supplement: S1 R Notebook — (HTML) [file pone.0227760.s002.html]

Interpreting condition number as the variance of noise


Code 

- Show All Code
- Hide All Code
- Download Rmd

# Interpreting condition number as the variance of noise

#### Trang Tran

#### 9/16/2019


\[
\DeclareMathOperator\*{\E}{\mathbb{E}}
\]

Condition-number based deviation (\(cdev\)) is defined to measure the difference of one expression matrix from another, given that they can be transformed into one another by a matrix.

Let \(A, B\) the matrices in question, and \(X\) the one that transform \(A\) into \(B\).

\[\begin{eqnarray\*}
A X &=& B \\
X &=& {A^{T}A}^{-1} A^T B \\
cdev(A,B) &=& \kappa(X)
\end{eqnarray\*}\]

If we define an ideal normalization to be any matrix that can be transformed into the true abundance matrix by multiplying with a constant factor, \(A\) and \(B\) are the same if \(X\) is of identity form, regardless of the exact values on its diagonal. A smaller condition number \(\kappa(X)\) indicates the closer \(X\) is to the identity form,

In the following notes, we illustrated that

1. Alternative definitions of condition number resulted in numerically identical values,
2. Condition number is proportional to the amount of noise added to the identity matrix

### Alternative definitions

There are two different definitions of condition number:

1. Ratio \(C\) of the largest to smallest singular values of a matrix (http://mathworld.wolfram.com/ConditionNumber.html)
2. \(C = \|X\| \cdot\|X^{-1}\|\), with \(\|\cdot\|\) being \(L^2\) norm.

The calculations below showed that the two definitions resulted in identical values of condition number.


```
cn.def1 <- function(X) {
    sigmas = svd(X,nu=0,nv=0) %>% 
        `[[`('d')
    return(sigmas[1] / sigmas[length(sigmas)])
}

cn.def2 <- function(X) { 
    return(norm(X,'2') %*% norm(MASS::ginv(X), '2'))
}
REPEATS = 100
tmp <- data.frame(cn.def1 = rep(0,REPEATS), cn.def2 = rep(0, REPEATS))
for(i in 1:REPEATS) {
    X = matrix(rnorm(100),nrow=10)
    tmp[i,'cn.def1'] <- cn.def1(X) 
    tmp[i,'cn.def2'] <- cn.def2(X) 
}
plot(tmp[,'cn.def1'], tmp[,'cn.def2'], log='xy',
     xlab = 'Definition 1', ylab = 'Definition 2')
```

### Condition number as a function of perturbation to Identity form

Three types of perturbation to identity matrix is simulated to observe the behavior of condition number.

1. Multiplying by constant factor
2. Adding random uniform noise
3. Adding random normal noise

It is observed that

1. Multiplying by constant factor does not alter the identify form, thus preserving condition number.
2. Condition number if proportional to variance of the random noise added to the identity matrix.
3. In the case of random normal noise, sufficiently small condition number can be a reliable indicator of the amount of noise.

#### Multiplication by constant factor does not alter the identify form, thus preserving condition number


```
I = diag(nrow=20)
tmp = data.frame('noise'= rep('const',10),
                 'alpha' = c(-1e4, -1e3,0, 0.5, 0.75, 1, 10, 2e2, 1e3, 1e4)
                 )
for (i in 1:nrow(tmp)) {
    X = tmp[i, 'alpha'] * I 
    tmp[i,'conditionNumber'] = cn.def1(X)
}
head(tmp)
```

#### Random uniform noise


```
I = diag(nrow=20)
tmp = data.frame('noise'= 'unif',
                 'b' = rexp(300, rate=2)
                 )
for (i in 1:nrow(tmp)) {
    X = I + matrix(runif(length(I),min=0, max=tmp[i,'b']),
                   nrow=nrow(I))
    tmp[i,'conditionNumber'] = cn.def1(X)
}
plot(tmp[,'conditionNumber'], tmp[,'b'], log='xy',
     xlab = TeX('$\\kappa(\\mathbf{I} + noise)$'),
     ylab = 'b',
     main = TeX('$noise \\sim Unif(0, b)$'))
```

#### Random normal noise


```
I = diag(nrow=20)
tmp = data.frame('noise'= 'normal',
                 'mean' = 0,
                 'sd'   = rexp(500, rate=2) 
                 )
for (i in 1:nrow(tmp)) {
    X = I + matrix(rnorm(length(I),mean=0, sd=tmp[i,'sd']),
                   nrow=nrow(I))
    tmp[i,'conditionNumber'] = cn.def1(X)
}
plot(tmp[,'conditionNumber'], tmp[,'sd'], log='x',
     xlab = TeX('$\\kappa(\\mathbf{I} + noise)$'),
     ylab = TeX('$\\sigma$'),
     main = TeX('$noise \\sim N(0, \\sigma^2)$'))
```

#### Sufficiently small condition number can predict the amount of noise

Condition number, if not too large, can predict the variance of the noise added to the Identity matrix. From the above examples, we can see that condition number can predict the variance of noise with good confidence up to \(C \approx 10\) in the case of random noise, and up to \(C \approx 100\) in the case of normal noise. Above these values, the confidence decrease quickly. To regress the amount of perturbation against C, it maybe desirable to disregard values on the higher ends.


```
plot(log(tmp$conditionNumber), tmp$sd, xlab=expression(log(C)), ylab=expression(sigma))
maxConds = c(1e8, 1e6, 1e4, 100, 50, 10)
fits = lapply(1:length(maxConds), function(i) {
    maxCond = maxConds[i]
    obs_weights = log(maxCond) - log(tmp$conditionNumber)
    obs_weights[obs_weights < 0] = 0
    fit <- MASS::rlm( log(tmp$conditionNumber), tmp$sd,weights=obs_weights)
    abline(0, fit$coefficients[[1]], col=i, lty=i)
    return(fit)
})
```


```
'rlm' failed to converge in 20 steps
```


```
legend("topright", "(x,y)", legend = maxConds, col=1:length(maxConds), lty = 1:length(maxConds))
```


For example, if condition number values above 10 are disregarded when fitting, the standard deviation of normal noise can be estimated using

\[
\hat{\sigma} = 0.084\log(C)
\]

LS0tCnRpdGxlOiAiSW50ZXJwcmV0aW5nIGNvbmRpdGlvbiBudW1iZXIgYXMgdGhlIHZhcmlhbmNlIG9mIG5vaXNlIgphdXRob3I6ICJUcmFuZyBUcmFuIgpkYXRlOiAiOS8xNi8yMDE5IgpvdXRwdXQ6IGh0bWxfbm90ZWJvb2sKLS0tCgpgYGB7ciBzZXR1cCwgaW5jbHVkZT1GQUxTRX0Ka25pdHI6Om9wdHNfY2h1bmskc2V0KGVjaG8gPSBUUlVFKQpsaWJyYXJ5KG1hZ3JpdHRyKQpsaWJyYXJ5KGdncGxvdDIpCmxpYnJhcnkobGF0ZXgyZXhwKQpgYGAKCiQkClxEZWNsYXJlTWF0aE9wZXJhdG9yKntcRX17XG1hdGhiYntFfX0KJCQKCkNvbmRpdGlvbi1udW1iZXIgYmFzZWQgZGV2aWF0aW9uICgkY2RldiQpIGlzIGRlZmluZWQgdG8gbWVhc3VyZSB0aGUgZGlmZmVyZW5jZSBvZiBvbmUgZXhwcmVzc2lvbiBtYXRyaXggZnJvbSBhbm90aGVyLCBnaXZlbiB0aGF0IHRoZXkgY2FuIGJlIHRyYW5zZm9ybWVkIGludG8gb25lIGFub3RoZXIgYnkgYSBtYXRyaXguIAoKTGV0ICRBLCBCJCB0aGUgbWF0cmljZXMgaW4gcXVlc3Rpb24sIGFuZCAkWCQgdGhlIG9uZSB0aGF0IHRyYW5zZm9ybSAkQSQgaW50byAkQiQuCgpcYmVnaW57ZXFuYXJyYXkqfQpBIFggJj0mIEIJXFwKWCAmPSYge0Fee1R9QX1eey0xfSBBXlQgQiBcXApjZGV2KEEsQikgJj0mIFxrYXBwYShYKQpcZW5ke2VxbmFycmF5Kn0KCklmIHdlIGRlZmluZSBhbiBpZGVhbCBub3JtYWxpemF0aW9uIHRvIGJlIGFueSBtYXRyaXggdGhhdCBjYW4gYmUgdHJhbnNmb3JtZWQgaW50byB0aGUgdHJ1ZSBhYnVuZGFuY2UgbWF0cml4IGJ5IG11bHRpcGx5aW5nIHdpdGggYSBjb25zdGFudCBmYWN0b3IsICRBJCBhbmQgJEIkIGFyZSB0aGUgc2FtZSBpZiAkWCQgaXMgb2YgaWRlbnRpdHkgZm9ybSwgcmVnYXJkbGVzcyBvZiB0aGUgZXhhY3QgdmFsdWVzIG9uIGl0cyBkaWFnb25hbC4gQSBzbWFsbGVyIGNvbmRpdGlvbiBudW1iZXIgJFxrYXBwYShYKSQgaW5kaWNhdGVzIHRoZSBjbG9zZXIgJFgkIGlzIHRvIHRoZSBpZGVudGl0eSBmb3JtLAoKSW4gdGhlIGZvbGxvd2luZyBub3Rlcywgd2UgaWxsdXN0cmF0ZWQgdGhhdAoKMS4gQWx0ZXJuYXRpdmUgZGVmaW5pdGlvbnMgb2YgY29uZGl0aW9uIG51bWJlciByZXN1bHRlZCBpbiBudW1lcmljYWxseSBpZGVudGljYWwgdmFsdWVzLAoyLiBDb25kaXRpb24gbnVtYmVyIGlzIHByb3BvcnRpb25hbCB0byB0aGUgYW1vdW50IG9mIG5vaXNlIGFkZGVkIHRvIHRoZSBpZGVudGl0eSBtYXRyaXgKCiMjIyBBbHRlcm5hdGl2ZSBkZWZpbml0aW9ucwoKVGhlcmUgYXJlIHR3byBkaWZmZXJlbnQgZGVmaW5pdGlvbnMgb2YgY29uZGl0aW9uIG51bWJlcjoKCjEuIFJhdGlvICRDJCBvZiB0aGUgbGFyZ2VzdCB0byBzbWFsbGVzdCBzaW5ndWxhciB2YWx1ZXMgb2YgYSBtYXRyaXggKGh0dHA6Ly9tYXRod29ybGQud29sZnJhbS5jb20vQ29uZGl0aW9uTnVtYmVyLmh0bWwpCjIuICRDID0gXHxYXHwgXGNkb3RcfFheey0xfVx8JCwgd2l0aCAkXHxcY2RvdFx8JCBiZWluZyAkTF4yJCBub3JtLgoKVGhlIGNhbGN1bGF0aW9ucyBiZWxvdyBzaG93ZWQgdGhhdCB0aGUgdHdvIGRlZmluaXRpb25zIHJlc3VsdGVkIGluIGlkZW50aWNhbCB2YWx1ZXMgb2YgY29uZGl0aW9uIG51bWJlci4KCmBgYHtyfQpjbi5kZWYxIDwtIGZ1bmN0aW9uKFgpIHsKICAgIHNpZ21hcyA9IHN2ZChYLG51PTAsbnY9MCkgJT4lIAogICAgICAgIGBbW2AoJ2QnKQogICAgcmV0dXJuKHNpZ21hc1sxXSAvIHNpZ21hc1tsZW5ndGgoc2lnbWFzKV0pCn0KCmNuLmRlZjIgPC0gZnVuY3Rpb24oWCkgeyAKICAgIHJldHVybihub3JtKFgsJzInKSAlKiUgbm9ybShNQVNTOjpnaW52KFgpLCAnMicpKQp9ClJFUEVBVFMgPSAxMDAKdG1wIDwtIGRhdGEuZnJhbWUoY24uZGVmMSA9IHJlcCgwLFJFUEVBVFMpLCBjbi5kZWYyID0gcmVwKDAsIFJFUEVBVFMpKQpmb3IoaSBpbiAxOlJFUEVBVFMpIHsKICAgIFggPSBtYXRyaXgocm5vcm0oMTAwKSxucm93PTEwKQogICAgdG1wW2ksJ2NuLmRlZjEnXSA8LSBjbi5kZWYxKFgpIAogICAgdG1wW2ksJ2NuLmRlZjInXSA8LSBjbi5kZWYyKFgpIAp9CnBsb3QodG1wWywnY24uZGVmMSddLCB0bXBbLCdjbi5kZWYyJ10sIGxvZz0neHknLAogICAgIHhsYWIgPSAnRGVmaW5pdGlvbiAxJywgeWxhYiA9ICdEZWZpbml0aW9uIDInKQpgYGAKCiMjIyBDb25kaXRpb24gbnVtYmVyIGFzIGEgZnVuY3Rpb24gb2YgcGVydHVyYmF0aW9uIHRvIElkZW50aXR5IGZvcm0KClRocmVlIHR5cGVzIG9mIHBlcnR1cmJhdGlvbiB0byBpZGVudGl0eSBtYXRyaXggaXMgc2ltdWxhdGVkIHRvIG9ic2VydmUgdGhlIGJlaGF2aW9yIG9mIGNvbmRpdGlvbiBudW1iZXIuCgoxLiBNdWx0aXBseWluZyBieSBjb25zdGFudCBmYWN0b3IKMi4gQWRkaW5nIHJhbmRvbSB1bmlmb3JtIG5vaXNlCjMuIEFkZGluZyByYW5kb20gbm9ybWFsIG5vaXNlCgpJdCBpcyBvYnNlcnZlZCB0aGF0CgoxLiBNdWx0aXBseWluZyBieSBjb25zdGFudCBmYWN0b3IgZG9lcyBub3QgYWx0ZXIgdGhlIGlkZW50aWZ5IGZvcm0sIHRodXMgcHJlc2VydmluZyBjb25kaXRpb24gbnVtYmVyLgoyLiBDb25kaXRpb24gbnVtYmVyIGlmIHByb3BvcnRpb25hbCB0byB2YXJpYW5jZSBvZiB0aGUgcmFuZG9tIG5vaXNlIGFkZGVkIHRvIHRoZSBpZGVudGl0eSBtYXRyaXguCjMuIEluIHRoZSBjYXNlIG9mIHJhbmRvbSBub3JtYWwgbm9pc2UsIHN1ZmZpY2llbnRseSBzbWFsbCBjb25kaXRpb24gbnVtYmVyIGNhbiBiZSBhIHJlbGlhYmxlIGluZGljYXRvciBvZiB0aGUgYW1vdW50IG9mIG5vaXNlLgoKIyMjIyBNdWx0aXBsaWNhdGlvbiBieSBjb25zdGFudCBmYWN0b3IgZG9lcyBub3QgYWx0ZXIgdGhlIGlkZW50aWZ5IGZvcm0sIHRodXMgcHJlc2VydmluZyBjb25kaXRpb24gbnVtYmVyCgpgYGB7cn0KSSA9IGRpYWcobnJvdz0yMCkKdG1wID0gZGF0YS5mcmFtZSgnbm9pc2UnPSByZXAoJ2NvbnN0JywxMCksCiAgICAgICAgICAgICAgICAgJ2FscGhhJyA9IGMoLTFlNCwgLTFlMywwLCAwLjUsIDAuNzUsIDEsIDEwLCAyZTIsIDFlMywgMWU0KQogICAgICAgICAgICAgICAgICkKZm9yIChpIGluIDE6bnJvdyh0bXApKSB7CiAgICBYID0gdG1wW2ksICdhbHBoYSddICogSSAKICAgIHRtcFtpLCdjb25kaXRpb25OdW1iZXInXSA9IGNuLmRlZjEoWCkKfQpoZWFkKHRtcCkKYGBgCgojIyMjIFJhbmRvbSB1bmlmb3JtIG5vaXNlCgpgYGB7cn0KSSA9IGRpYWcobnJvdz0yMCkKdG1wID0gZGF0YS5mcmFtZSgnbm9pc2UnPSAndW5pZicsCiAgICAgICAgICAgICAgICAgJ2InID0gcmV4cCgzMDAsIHJhdGU9MikKICAgICAgICAgICAgICAgICApCmZvciAoaSBpbiAxOm5yb3codG1wKSkgewogICAgWCA9IEkgKyBtYXRyaXgocnVuaWYobGVuZ3RoKEkpLG1pbj0wLCBtYXg9dG1wW2ksJ2InXSksCiAgICAgICAgICAgICAgICAgICBucm93PW5yb3coSSkpCiAgICB0bXBbaSwnY29uZGl0aW9uTnVtYmVyJ10gPSBjbi5kZWYxKFgpCn0KcGxvdCh0bXBbLCdjb25kaXRpb25OdW1iZXInXSwgdG1wWywnYiddLCBsb2c9J3h5JywKICAgICB4bGFiID0gVGVYKCckXFxrYXBwYShcXG1hdGhiZntJfSArIG5vaXNlKSQnKSwKICAgICB5bGFiID0gJ2InLAogICAgIG1haW4gPSBUZVgoJyRub2lzZSBcXHNpbSBVbmlmKDAsIGIpJCcpKQpgYGAKCiMjIyMgUmFuZG9tIG5vcm1hbCBub2lzZQoKYGBge3J9CkkgPSBkaWFnKG5yb3c9MjApCnRtcCA9IGRhdGEuZnJhbWUoJ25vaXNlJz0gJ25vcm1hbCcsCiAgICAgICAgICAgICAgICAgJ21lYW4nID0gMCwKICAgICAgICAgICAgICAgICAnc2QnICAgPSByZXhwKDUwMCwgcmF0ZT0yKSAKICAgICAgICAgICAgICAgICApCmZvciAoaSBpbiAxOm5yb3codG1wKSkgewogICAgWCA9IEkgKyBtYXRyaXgocm5vcm0obGVuZ3RoKEkpLG1lYW49MCwgc2Q9dG1wW2ksJ3NkJ10pLAogICAgICAgICAgICAgICAgICAgbnJvdz1ucm93KEkpKQogICAgdG1wW2ksJ2NvbmRpdGlvbk51bWJlciddID0gY24uZGVmMShYKQp9CnBsb3QodG1wWywnY29uZGl0aW9uTnVtYmVyJ10sIHRtcFssJ3NkJ10sIGxvZz0neCcsCiAgICAgeGxhYiA9IFRlWCgnJFxca2FwcGEoXFxtYXRoYmZ7SX0gKyBub2lzZSkkJyksCiAgICAgeWxhYiA9IFRlWCgnJFxcc2lnbWEkJyksCiAgICAgbWFpbiA9IFRlWCgnJG5vaXNlIFxcc2ltIE4oMCwgXFxzaWdtYV4yKSQnKSkKYGBgCgojIyMjIFN1ZmZpY2llbnRseSBzbWFsbCBjb25kaXRpb24gbnVtYmVyIGNhbiBwcmVkaWN0IHRoZSBhbW91bnQgb2Ygbm9pc2UKCkNvbmRpdGlvbiBudW1iZXIsIGlmIG5vdCB0b28gbGFyZ2UsIGNhbiBwcmVkaWN0IHRoZSB2YXJpYW5jZSBvZiB0aGUgbm9pc2UgYWRkZWQgdG8gdGhlIElkZW50aXR5IG1hdHJpeC4gRnJvbSB0aGUgYWJvdmUgZXhhbXBsZXMsIHdlIGNhbiBzZWUgdGhhdCBjb25kaXRpb24gbnVtYmVyIGNhbiBwcmVkaWN0IHRoZSB2YXJpYW5jZSBvZiBub2lzZSB3aXRoIGdvb2QgY29uZmlkZW5jZSB1cCB0byAkQyBcYXBwcm94IDEwJCBpbiB0aGUgY2FzZSBvZiByYW5kb20gbm9pc2UsIGFuZCB1cCB0byAkQyBcYXBwcm94IDEwMCQgaW4gdGhlIGNhc2Ugb2Ygbm9ybWFsIG5vaXNlLiBBYm92ZSB0aGVzZSB2YWx1ZXMsIHRoZSBjb25maWRlbmNlIGRlY3JlYXNlIHF1aWNrbHkuIFRvIHJlZ3Jlc3MgdGhlIGFtb3VudCBvZiBwZXJ0dXJiYXRpb24gYWdhaW5zdCBDLCBpdCBtYXliZSBkZXNpcmFibGUgdG8gZGlzcmVnYXJkIHZhbHVlcyBvbiB0aGUgaGlnaGVyIGVuZHMuCgoKYGBge3J9CnBsb3QobG9nKHRtcCRjb25kaXRpb25OdW1iZXIpLCB0bXAkc2QsIHhsYWI9ZXhwcmVzc2lvbihsb2coQykpLCB5bGFiPWV4cHJlc3Npb24oc2lnbWEpKQptYXhDb25kcyA9IGMoMWU4LCAxZTYsIDFlNCwgMTAwLCA1MCwgMTApCmZpdHMgPSBsYXBwbHkoMTpsZW5ndGgobWF4Q29uZHMpLCBmdW5jdGlvbihpKSB7CiAgICBtYXhDb25kID0gbWF4Q29uZHNbaV0KICAgIG9ic193ZWlnaHRzID0gbG9nKG1heENvbmQpIC0gbG9nKHRtcCRjb25kaXRpb25OdW1iZXIpCiAgICBvYnNfd2VpZ2h0c1tvYnNfd2VpZ2h0cyA8IDBdID0gMAogICAgZml0IDwtIE1BU1M6OnJsbSggbG9nKHRtcCRjb25kaXRpb25OdW1iZXIpLCB0bXAkc2Qsd2VpZ2h0cz1vYnNfd2VpZ2h0cykKICAgIGFibGluZSgwLCBmaXQkY29lZmZpY2llbnRzW1sxXV0sIGNvbD1pLCBsdHk9aSkKICAgIHJldHVybihmaXQpCn0pCgpsZWdlbmQoInRvcHJpZ2h0IiwgIih4LHkpIiwgbGVnZW5kID0gbWF4Q29uZHMsIGNvbD0xOmxlbmd0aChtYXhDb25kcyksIGx0eSA9IDE6bGVuZ3RoKG1heENvbmRzKSkKYGBgCgpGb3IgZXhhbXBsZSwgaWYgY29uZGl0aW9uIG51bWJlciB2YWx1ZXMgYWJvdmUgMTAgYXJlIGRpc3JlZ2FyZGVkIHdoZW4gZml0dGluZywgdGhlIHN0YW5kYXJkIGRldmlhdGlvbiBvZiBub3JtYWwgbm9pc2UgY2FuIGJlIGVzdGltYXRlZCB1c2luZwoKJCQKXGhhdHtcc2lnbWF9ID0gMC4wODRcbG9nKEMpCiQkCgoK
