# Supplementary material for: A graph-based algorithm for RNA-seq data normalization
Source: PLoS One. 2020 Jan 24;15(1):e0227760. doi: 10.1371/journal.pone.0227760 (PMC6980396; doi:10.1371/journal.pone.0227760)
Supplement: S2 R Notebook — (HTML) [file pone.0227760.s003.html]

Effect of normalization on batch effect correction


Code 

- Show All Code
- Hide All Code
- Download Rmd

# Effect of normalization on batch effect correction

- Prepare data set
- Normalization methods
  - Pairwise condition-number-based deviations
- PCA
  - Before batch correction
  - After batch correction with combat
- Sample clustering
  - Before batch correction
    - Heatmap using Euclidean distance
    - Heatmap using correlation
  - After batch correction with combat
    - Heatmap using Euclidean distance
    - Heatmap using correlation
    - Varying clustering algorithms and parameters
      - Hierarchical clustering
      - K-means
      - Spectral clustering
    - Similarity with tissue-based clusters
- References


## Prepare data set

The data were provided as companion to the re-analysis by Gilad and Mizrahi-Man (Gilad and Mizrahi-Man 2015), at doi:10.5281/zenodo.17606 (Mizrahi-Man and Gilad 2015).


```
geneDetails = data.table::fread('../data/Gilad2015/R_input_files/ortholog_GC_table.txt')
stanford.meta = data.table::fread('../data/Gilad2015/R_input_files/Stanford_datasets.txt',header=FALSE) %>%
    set_names(c('label', 'sequence_identifier', 'species', 'tissue'))
stanford.fpkm = data.table::fread('../data/Gilad2015/R_input_files/Stanford_datasets_fpkmMat.txt') %>%
    t() %>%
    set_colnames(geneDetails$human_name) %>%
    set_rownames(stanford.meta$label) %>%
    subset(., TRUE, !grepl('mt-', x = geneDetails$mouse_name)) %>%
    remove_invariant_genes()
stanford.cnt = data.table::fread('../data/Gilad2015/R_input_files/Stanford_datasets_rawCountsMat.txt') %>%
    t() %>%
    set_colnames(geneDetails$human_name) %>%
    set_rownames(stanford.meta$label) %>%
    subset(., TRUE, !grepl('mt-', x = geneDetails$mouse_name)) %>%
    remove_invariant_genes() 
stanford.cnt %>%
    remove_lowest_quantile(p = 0.3) %>%
    project.pca(selected.pc = c(1,2)) %>%
    cbind(stanford.meta) %>%
    plot_pca(shape = 'tissue', color = 'species')
```


```
stanford.cnt %>%
    remove_lowest_quantile(p = 0.3) %>%
    project.pca(selected.pc = c(1,2), scale = TRUE) %>%
    cbind(stanford.meta) %>%
    plot_pca(shape = 'tissue', color = 'species')
```

## Normalization methods

The following normalization procedures are examined in this analysis:

- No scaling (raw)
- Total Count (TC)
- Upper Quantile (UQ) (Bullard et al. 2010)
- TMM (Robinson and Oshlack 2010)
- DESeq (Anders and Huber 2010)
- PoissonSeq (Li et al. 2012)
- Graph-based normalization (gbnorm) (described in this manuscript)


```
methods = list('raw' = NA,
               'TC' = NA,
               'UQ' = gbnorm::normalize.by.uq,
               'TMM' = gbnorm::normalize.by.tmm,
               'DESeq' = gbnorm::normalize.by.deseq,
               'PoissonSeq' = gbnorm::normalize.by.poissonseq,
               'gbnorm' = NA)
methods$raw <- function(X) {
    return(X)
}


methods$TC <- function(X) {
    scaleFactors = apply(X, 1, sum)
    scaleFactors = scaleFactors / geom.mean(scaleFactors)
    return(sweep(X, 1, STATS = scaleFactors, FUN = "/"))
}

methods$gbnorm <- function(X) {
    refs = gbnorm::get.references.apcluster(X,
                                   cor.method = 'pearson',
                                   min.count = 10) %>%
        `$`('Name')
    X.normed = which(colnames(X) %in% refs) %>%
        gbnorm::normalize.by.refs(X, ., scale = TRUE)
    return(X.normed)
}
```


```
norm.stanford <- mclapply(1:length(methods), mc.cores = min(4, length(methods), detectCores() - 1), FUN = function(i) {
    start = proc.time()
    X.normed <- do.call(methods[[i]], list(stanford.cnt))
    dt = (proc.time() - start)['elapsed']
    return(list('method' = names(methods)[[i]],
                'time' = dt,
                'normalizedCounts' = X.normed))
}) %>%
    set_names(names(methods))
```


```
saveRDS(norm.stanford, file = 'stanford-normalized.RDS')
```


```
# Pre-computed normalizations are loaded, in case they are available
norm.stanford = readRDS('stanford-normalized.RDS')
```


### Pairwise condition-number-based deviations

\(cdev\) between every pair of expression matrices is calculated below. All normalized count matrices are close to one another but differ significantly from the raw count matrix.


```
mclapply(names(norm.stanford), mc.cores = min(length(norm.stanford), detectCores() - 2), FUN = function(i) {
    sapply(names(norm.stanford), function(j) {
        gbnorm::cdev(norm.stanford[[i]]$normalizedCounts, norm.stanford[[j]]$normalizedCounts) %>%
            return()
    })
}) %>%
    do.call(rbind, .) %>%
    set_rownames(names(norm.stanford)) %>%
    set_colnames(names(norm.stanford))
```


```
                raw        TC        UQ       TMM
raw         1.00000 10.816567 17.183206 14.413205
TC         10.81657  1.000000  2.535467  2.870660
UQ         17.18321  2.535467  1.000000  1.756399
TMM        14.41321  2.870660  1.756399  1.000000
DESeq      18.46971  2.667187  1.626987  1.475262
PoissonSeq 17.32187  2.632646  1.285957  2.066885
gbnorm     21.72567  5.171068  3.744616  4.580364
               DESeq PoissonSeq    gbnorm
raw        18.469710  17.321875 21.725674
TC          2.667187   2.632646  5.171068
UQ          1.626987   1.285957  3.744616
TMM         1.475262   2.066885  4.580364
DESeq       1.000000   1.885216  4.743949
PoissonSeq  1.885216   1.000000  3.161671
gbnorm      4.743949   3.161671  1.000000
```

## PCA

### Before batch correction


```
for (i in names(norm.stanford)) {
    x = norm.stanford[[i]]$normalizedCounts
    p = remove_lowest_quantile(x, p = 0.3) %>%
        project.pca(selected.pc = c(1,2),center = TRUE, scale = TRUE) %>%
        cbind(stanford.meta) %>%
        plot_pca(shape = 'tissue', color = 'species')   +
        ggtitle(i) + 
        guides(shape=guide_legend(ncol = 2))
    print(p)
}
```


```
for (i in names(norm.stanford)) {
    x = norm.stanford[[i]]$normalizedCounts
    p = remove_lowest_quantile(x, p = 0.3) %>%
        project.pca(selected.pc = c(1,2),center = TRUE, scale = TRUE) %>%
        cbind(stanford.meta) %>%
        plot_pca(shape = 'sequence_identifier', color = 'species')   +
        ggtitle(i)
    print(p)
}
```

### After batch correction with combat


```
norm.combat.stanford = lapply(names(norm.stanford), function(i) {
    x = norm.stanford[[i]]$normalizedCounts %>%
        add(1) %>%
        log2() %>%
        t() %>%
        sva::ComBat(batch = stanford.meta$sequence_identifier,
                    mod = model.matrix(~1, data = data.frame(tissue = stanford.meta$tissue, species = stanford.meta$species)),
                    par.prior = TRUE) %>%
        t()
    return(x)
}) %>%
    set_names(names(norm.stanford))
```


```
Found 1242 genes with uniform expression within a single batch (all zeros); these will not be adjusted for batch.
```


```
Found5batches
Adjusting for0covariate(s) or covariate level(s)
```


```
Standardizing Data across genes
```


```
Fitting L/S model and finding priors
Finding parametric adjustments
Adjusting the Data
```


```
Found 1242 genes with uniform expression within a single batch (all zeros); these will not be adjusted for batch.
```


```
Found5batches
Adjusting for0covariate(s) or covariate level(s)
```


```
Standardizing Data across genes
```


```
Fitting L/S model and finding priors
Finding parametric adjustments
Adjusting the Data
```


```
Found 1242 genes with uniform expression within a single batch (all zeros); these will not be adjusted for batch.
```


```
Found5batches
Adjusting for0covariate(s) or covariate level(s)
```


```
Standardizing Data across genes
```


```
Fitting L/S model and finding priors
Finding parametric adjustments
Adjusting the Data
```


```
Found 1242 genes with uniform expression within a single batch (all zeros); these will not be adjusted for batch.
```


```
Found5batches
Adjusting for0covariate(s) or covariate level(s)
```


```
Standardizing Data across genes
```


```
Fitting L/S model and finding priors
Finding parametric adjustments
Adjusting the Data
```


```
Found 1242 genes with uniform expression within a single batch (all zeros); these will not be adjusted for batch.
```


```
Found5batches
Adjusting for0covariate(s) or covariate level(s)
```


```
Standardizing Data across genes
```


```
Fitting L/S model and finding priors
Finding parametric adjustments
Adjusting the Data
```


```
Found 1242 genes with uniform expression within a single batch (all zeros); these will not be adjusted for batch.
```


```
Found5batches
Adjusting for0covariate(s) or covariate level(s)
```


```
Standardizing Data across genes
```


```
Fitting L/S model and finding priors
Finding parametric adjustments
Adjusting the Data
```


```
Found 1242 genes with uniform expression within a single batch (all zeros); these will not be adjusted for batch.
```


```
Found5batches
Adjusting for0covariate(s) or covariate level(s)
```


```
Standardizing Data across genes
```


```
Fitting L/S model and finding priors
Finding parametric adjustments
Adjusting the Data
```


```
for (i in names(norm.combat.stanford)) {
    x = norm.combat.stanford[[i]]
    p = remove_lowest_quantile(x, p = 0.3) %>%
        project.pca(selected.pc = c(1,2),center = TRUE, scale = TRUE) %>%
        cbind(stanford.meta) %>%
        plot_pca(shape = 'tissue', color = 'species')   +
        ggtitle(i)
    print(p)
}
```


```
for (i in names(norm.combat.stanford)) {
    x = norm.combat.stanford[[i]]
    p = remove_lowest_quantile(x, p = 0.3) %>%
        project.pca(selected.pc = c(1,2),center = TRUE, scale = TRUE) %>%
        cbind(stanford.meta) %>%
        plot_pca(shape = 'species', color = 'sequence_identifier')   +
        ggtitle(i)
    print(p)
}
```

## Sample clustering

### Before batch correction


```
hclust.euclid.stanford = lapply(names(norm.stanford), function(i) {
    x = norm.stanford[[i]]$normalizedCounts
    remove_lowest_quantile(x) %>%
        dist(method = 'euclidean') %>%
        hclust()
}) %>%
    set_names(names(norm.stanford))
sapply(hclust.euclid.stanford, function(x) {
    cutree(x, k = 13) %>%
    mclust::adjustedRandIndex(stanford.meta$tissue)
}) %>%
    set_names(names(hclust.euclid.stanford))
```


```
       raw         TC         UQ        TMM      DESeq 
0.02049427 0.08376963 0.02812242 0.02812242 0.02812242 
PoissonSeq     gbnorm 
0.03982930 0.05063291
```


```
lapply(names(norm.stanford), function(i) {
    x = norm.stanford[[i]]$normalizedCounts
    remove_lowest_quantile(x) %>%
        dist(method = 'euclidean') %>%
        hclust(method = 'average')
}) %>%
    set_names(names(norm.stanford)) %>%
    sapply(function(x) {
    cutree(x, k = 13) %>%
    mclust::adjustedRandIndex(stanford.meta$tissue)
}) %>%
    set_names(names(norm.stanford))
```


```
        raw          TC          UQ         TMM 
0.007444169 0.066293184 0.028122415 0.028122415 
      DESeq  PoissonSeq      gbnorm 
0.028122415 0.066293184 0.060349391
```


```
lapply(names(norm.stanford), function(i) {
    x = norm.stanford[[i]]$normalizedCounts
    remove_lowest_quantile(x) %>%
        dist(method = 'euclidean') %>%
        hclust(method = 'average')
}) %>%
    set_names(names(norm.stanford)) %>%
    sapply(function(x) {
    cutree(x, k = 13) %>%
    mclust::adjustedRandIndex(stanford.meta$tissue)
}) %>%
    set_names(names(norm.stanford))
```


```
        raw          TC          UQ         TMM 
0.007444169 0.066293184 0.028122415 0.028122415 
      DESeq  PoissonSeq      gbnorm 
0.028122415 0.066293184 0.060349391
```


```
lapply(names(norm.stanford), function(i) {
    x = norm.stanford[[i]]$normalizedCounts
    remove_lowest_quantile(x) %>%
        dist(method = 'manhattan') %>%
        hclust(method = 'complete')
}) %>%
    set_names(names(norm.stanford)) %>%
    sapply(function(x) {
    cutree(x, k = 13) %>%
    mclust::adjustedRandIndex(stanford.meta$tissue)
}) %>%
    set_names(names(norm.stanford))
```


```
         raw           TC           UQ          TMM 
-0.008064516 -0.059730250 -0.035502959 -0.061946903 
       DESeq   PoissonSeq       gbnorm 
-0.068090788 -0.035502959 -0.031294452
```


```
lapply(names(norm.stanford), function(i) {
    x = norm.stanford[[i]]$normalizedCounts
    remove_lowest_quantile(x) %>%
        dist(method = 'manhattan') %>%
        hclust(method = 'average')
}) %>%
    set_names(names(norm.stanford)) %>%
    sapply(function(x) {
    cutree(x, k = 13) %>%
    mclust::adjustedRandIndex(stanford.meta$tissue)
}) %>%
    set_names(names(norm.stanford))
```


```
        raw          TC          UQ         TMM 
-0.03550296 -0.06749311 -0.03773585 -0.01273148 
      DESeq  PoissonSeq      gbnorm 
-0.03773585 -0.03773585 -0.01987448
```


K-means


```
lapply(names(norm.stanford), function(i) {
    x = norm.stanford[[i]]$normalizedCounts
    remove_lowest_quantile(x) %>%
        kmeans(centers = 13, iter.max = 30)
}) %>% 
    set_names(names(norm.stanford)) %>%
    sapply(function(x) {
        x$cluster %>%
        mclust::adjustedRandIndex(stanford.meta$tissue)
    }) %>%
    set_names(names(norm.stanford))
```


```
        raw          TC          UQ         TMM 
0.056224900 0.119241192 0.039829303 0.004267425 
      DESeq  PoissonSeq      gbnorm 
0.056224900 0.148606811 0.019024970
```


#### Heatmap using Euclidean distance


```
bluePallette = RColorBrewer::brewer.pal(9, 'Blues')
blue255 = colorRampPalette(rev(bluePallette))(255)
pal2 = RColorBrewer::brewer.pal(2, 'Accent')[1:2] %>%
    set_names(unique(stanford.meta$species))
```


```
minimal value for n is 3, returning requested palette with 3 different levels
```


```
pal5 = RColorBrewer::brewer.pal(5, 'Accent') %>%
    set_names(unique(stanford.meta$sequence_identifier))
species_colors = list('species' = pal2,
                      'batch' = pal5)

for (i in names(norm.stanford)) {
    x = norm.stanford[[i]]$normalizedCounts
    dist.x = remove_lowest_quantile(x, p = 0.3) %>%
        dist(method = 'euclidean')
    hclust.x = hclust(dist.x, method = 'average')
    pheatmap::pheatmap(as.matrix(dist.x),
                       # clustering_distance_rows = hclust.x, clustering_distance_cols = hclust.x,
                       cluster_rows = hclust.x, cluster_cols = hclust.x,
                       labels_row = stanford.meta$label, labels_col = stanford.meta$sequence_identifier,
                       annotation_row = data.frame('species' = stanford.meta$species, row.names = stanford.meta$label),
                       annotation_col = data.frame('batch' = stanford.meta$sequence_identifier, row.names = stanford.meta$label),
                       annotation_colors = species_colors,
                       cellwidth = 12, cellheight = 12,main = sprintf('%s', i),
                        col = blue255)
}
```

#### Heatmap using correlation

Clustering using correlation as similarity metrics are identical, since global scaling does not affect correlation between samples.


```
for (i in names(norm.stanford)) {
    x = norm.stanford[[i]]$normalizedCounts
    cor(t(x), method = 'pearson') %>%
        pheatmap::pheatmap(clustering_distance_rows = 'correlation', clustering_distance_cols = 'correlation',
                           clustering_method = 'single',
                           annotation_row = data.frame('species' = stanford.meta$species, row.names = stanford.meta$label),
                           annotation_col = data.frame('batch' = stanford.meta$sequence_identifier, row.names = stanford.meta$label),
                           annotation_colors = species_colors,
                           labels_row = stanford.meta$label, labels_col = stanford.meta$sequence_identifier,
                           cellwidth = 12, cellheight = 12, main = 'No batch correction')
}
```

### After batch correction with combat

#### Heatmap using Euclidean distance


```
for (i in names(norm.combat.stanford)) {
    x = norm.combat.stanford[[i]]
    dist.x = remove_lowest_quantile(x, p = 0.3) %>%
        dist(method = 'euclidean')
    hclust.x = hclust(dist.x, method = 'average')
    pheatmap::pheatmap(as.matrix(dist.x),
                       cluster_rows = hclust.x, cluster_cols = hclust.x,
                       annotation_row = data.frame('species' = stanford.meta$species, row.names = stanford.meta$label),
                       annotation_col = data.frame('batch' = stanford.meta$sequence_identifier, row.names = stanford.meta$label),
                       annotation_colors = species_colors,
                       labels_row = stanford.meta$label, labels_col = stanford.meta$sequence_identifier,
                       cellwidth = 12, cellheight = 12,main = sprintf('%s + combat', i),
                        col = blue255)
}
```

#### Heatmap using correlation


```
for (i in names(norm.combat.stanford)) {
    x = norm.combat.stanford[[i]] %>%
        remove_lowest_quantile(p = 0.3)
    cor(t(x), method = 'pearson') %>%
        pheatmap::pheatmap(clustering_distance_rows = 'correlation', clustering_distance_cols = 'correlation',
                           clustering_method = 'single',
                           annotation_row = data.frame('species' = stanford.meta$species, row.names = stanford.meta$label),
                           annotation_col = data.frame('batch' = stanford.meta$sequence_identifier, row.names = stanford.meta$label),
                           annotation_colors = species_colors,
                           labels_row = stanford.meta$label, labels_col = stanford.meta$sequence_identifier,
                           cellwidth = 12, cellheight = 12, main = sprintf('%s + combat', i))
}
```

#### Varying clustering algorithms and parameters

Normalizing read counts differently before batch effect correction resulted in different clustering patterns. To compare these patterns, we chose, rather arbitrarily, the grouping that aligns with tissue types, as the reference, and quantify the similarity of a clustering pattern with this reference by adjusted rand index. A higher adjusted rand index indicates more similar clustering patterns.

Assuming that the grouping of samples by their tissue of origin (rather than by species) is a desirable clustering pattern, a higher adjusted rand index therefore indicates a better processed input. Although this assumption may not be 100% accurate on the current data set, given the low number of replicates and high level of variation in this data set (Chan et al. 2009), it is guided by the conservation of tissue transcriptomic profiles observed across many vertebrate species, using both microarray (Chan et al. 2009) and RNA-seq (Sudmant, Alexis, and Burge 2015 and the RNA-seq studies therein).

Since the choice of distance metrics, clustering algorithm and clustering parameters all lead to different outcomes, we performed clustering at various algorithms and parameters, in order to see if the differences among those clustering patterns remain stable. For K-means and spectral clustering in which the centers were initialized randomly, clustering was repeated multiple times and the all the results were recorded for comparison.

For each clustering result, an adjusted rand index (ARI) with respect to reference (i.e. tissue-based) clusters was calculated, and used to rank the normalization method associated with it, low ARI corresponds to low rank. Raw counts (`raw + combat`) consistently rank at the bottom, practically not affected by algorithmic and parametric choices of clustering. The differences in ranking between normalization methods are less clear and less consistent.

##### Hierarchical clustering


```
clust.norm.combat = list()
```


```
hl = lapply(names(norm.combat.stanford), function(i) {
    x = norm.combat.stanford[[i]]
    remove_lowest_quantile(x) %>%
        dist(method = 'euclidean') %>%
        hclust()
}) %>%
    set_names(names(norm.combat.stanford)) %>%
    sapply(function(x) {
    cutree(x, k = 13) %>%
    mclust::adjustedRandIndex(stanford.meta$tissue)
}) %>%
    set_names(names(norm.combat.stanford)) %>%
    list('result' = .,
         'algo' = 'Hierarchical clustering',
         'R function' = 'hclust',
         'parameters' = 'method=complete;dist=euclidean')
clust.norm.combat = append(clust.norm.combat, list(hl))
hl$result
```


```
       raw         TC         UQ        TMM      DESeq 
0.07643312 0.31930693 0.30167598 0.30167598 0.30167598 
PoissonSeq     gbnorm 
0.30167598 0.31930693
```


```
hl = lapply(names(norm.combat.stanford), function(i) {
    x = norm.combat.stanford[[i]]
    remove_lowest_quantile(x) %>%
        dist(method = 'euclidean') %>%
        hclust(method = 'average')
}) %>%
    set_names(names(norm.combat.stanford)) %>%
    sapply(function(x) {
    cutree(x, k = 13) %>%
    mclust::adjustedRandIndex(stanford.meta$tissue)
}) %>%
    set_names(names(norm.combat.stanford)) %>%
    list('result' = .,
         'algo' = 'Hierarchical clustering',
         'R function' = 'hclust',
         'parameters' = 'method=average;dist=euclidean')
clust.norm.combat = append(clust.norm.combat, list(hl))
```


```
hl = lapply(names(norm.combat.stanford), function(i) {
    x = norm.combat.stanford[[i]]
    remove_lowest_quantile(x) %>%
        dist(method = 'manhattan') %>%
        hclust(method = 'complete')
}) %>%
    set_names(names(norm.combat.stanford)) %>%
    sapply(function(x) {
    cutree(x, k = 13) %>%
    mclust::adjustedRandIndex(stanford.meta$tissue)
}) %>%
    set_names(names(norm.combat.stanford))  %>%
    list('result' = .,
         'algo' = 'Hierarchical clustering',
         'R function' = 'hclust',
         'parameters' = 'method=complete;dist=manhanttan')
clust.norm.combat = append(clust.norm.combat, list(hl))
```


```
hl = lapply(names(norm.combat.stanford), function(i) {
    x = norm.combat.stanford[[i]]
    remove_lowest_quantile(x) %>%
        dist(method = 'manhattan') %>%
        hclust(method = 'average')
}) %>%
    set_names(names(norm.combat.stanford)) %>%
    sapply(function(x) {
    cutree(x, k = 13) %>%
    mclust::adjustedRandIndex(stanford.meta$tissue)
}) %>%
    set_names(names(norm.combat.stanford))  %>%
    list('result' = .,
         'algo' = 'Hierarchical clustering',
         'R function' = 'hclust',
         'parameters' = 'method=average;dist=manhanttan')
clust.norm.combat = append(clust.norm.combat, list(hl))
hl$result
```


```
        raw          TC          UQ         TMM 
0.004683841 0.180327869 0.257425743 0.222222222 
      DESeq  PoissonSeq      gbnorm 
0.222222222 0.257425743 0.230330673
```


```
hl = lapply(names(norm.combat.stanford), function(i) {
    x = norm.combat.stanford[[i]] %>%
        remove_lowest_quantile(p = 0.3)
    (1 - cor(t(x))) %>%
        as.dist() %>%
        hclust(method = 'single')
}) %>%
    set_names(names(norm.combat.stanford)) %>%
    sapply(function(x) {
    cutree(x, k = 13) %>%
    mclust::adjustedRandIndex(stanford.meta$tissue)
}) %>%
    set_names(names(norm.combat.stanford)) %>%
    list('result' = .,
         'algo' = 'Hierarchical clustering',
         'R function' = 'hclust',
         'parameters' = 'method=single;dist=1-cor')
clust.norm.combat = append(clust.norm.combat, list(hl))
hl$result
```


```
       raw         TC         UQ        TMM      DESeq 
 0.1109531  0.2224317  0.2224317  0.2224317  0.2224317 
PoissonSeq     gbnorm 
 0.2224317  0.2224317
```

##### K-means


```
N_REPEATS = 20
cl = lapply(names(norm.combat.stanford), function(i) {
    x = norm.combat.stanford[[i]] %>%
        remove_lowest_quantile(p = 0.3)
    lapply(1:N_REPEATS, function(j) {
        kl = kmeans(x, centers = 13, iter.max = 50)
        return(kl$cluster)
    })
}) %>%
    set_names(names(norm.combat.stanford)) %>%
    lapply(function(x) {
        sapply(x, function(x_j) {
            mclust::adjustedRandIndex(x_j, stanford.meta$tissue)
        })
    }) %>%
    set_names(names(norm.combat.stanford))  %>%
    do.call(rbind, .) %>%
    list('result' = .,
         'algo' = 'K-means',
         'R function' = 'stats::kmeans',
         'parameters' = 'method=Hatigan-Wong; k = 13')
clust.norm.combat = append(clust.norm.combat, list(cl))
cl$result
```


```
                [,1]        [,2]       [,3]      [,4]
raw        0.1620112 -0.00422833 0.09217877 0.2318436
TC         0.4248985  0.31457431 0.37150838 0.3572395
UQ         0.3572395  0.35723951 0.35723951 0.3016760
TMM        0.3572395  0.27821522 0.38118812 0.3572395
DESeq      0.3438320  0.35723951 0.28958051 0.4248985
PoissonSeq 0.4248985  0.22192152 0.35723951 0.2219215
gbnorm     0.3016760  0.34383202 0.20382166 0.3572395
                 [,5]       [,6]      [,7]      [,8]
raw        0.07643312 0.07643312 0.1620112 0.1620112
TC         0.23184358 0.37150838 0.3572395 0.3572395
UQ         0.24789410 0.22192152 0.3715084 0.2782152
TMM        0.42489851 0.37150838 0.4248985 0.3016760
DESeq      0.28958051 0.35723951 0.3016760 0.3016760
PoissonSeq 0.35723951 0.30167598 0.2895805 0.2895805
gbnorm     0.31457431 0.20382166 0.4248985 0.2895805
                [,9]      [,10]      [,11]      [,12]
raw        0.2318436 0.08660352 0.08660352 0.07643312
TC         0.2478941 0.35723951 0.42489851 0.35723951
UQ         0.3572395 0.35723951 0.42489851 0.35723951
TMM        0.2219215 0.35723951 0.25742574 0.28958051
DESeq      0.3016760 0.28958051 0.30167598 0.28958051
PoissonSeq 0.3572395 0.35723951 0.28958051 0.37150838
gbnorm     0.3438320 0.28958051 0.37150838 0.25742574
                [,13]     [,14]     [,15]      [,16]
raw        0.09217877 0.1620112 0.2318436 0.09217877
TC         0.35723951 0.3572395 0.3572395 0.37150838
UQ         0.35723951 0.3572395 0.3572395 0.35723951
TMM        0.42489851 0.2895805 0.2895805 0.40944882
DESeq      0.42489851 0.3016760 0.3572395 0.35723951
PoissonSeq 0.30167598 0.2895805 0.3715084 0.42489851
gbnorm     0.35723951 0.3145743 0.3715084 0.35723951
               [,17]      [,18]     [,19]     [,20]
raw        0.1620112 0.07178218 0.2318436 0.1620112
TC         0.4248985 0.28958051 0.2895805 0.3572395
UQ         0.3572395 0.35723951 0.3572395 0.4248985
TMM        0.2782152 0.42489851 0.3016760 0.3572395
DESeq      0.4248985 0.28958051 0.3572395 0.3572395
PoissonSeq 0.3715084 0.35723951 0.3572395 0.3572395
gbnorm     0.1469816 0.20382166 0.3572395 0.3715084
```

##### Spectral clustering


```
N_REPEATS = 20
sc = lapply(names(norm.combat.stanford), function(i) {
    x = norm.combat.stanford[[i]] %>%
        remove_lowest_quantile(p = 0.3)
    lapply(1:N_REPEATS, function(j) {
        return(kernlab::specc(x, centers = 13))
    })
}) %>% 
    set_names(names(norm.combat.stanford)) %>%
    lapply(function(x) {
        sapply(x, function(x_j) {
            as.numeric(x_j) %>%
                mclust::adjustedRandIndex(stanford.meta$tissue)
        })        
    }) %>%
    set_names(names(norm.combat.stanford))  %>%
    do.call(rbind, .) %>%
    list('result' = .,
         'algo' = 'Spectral clustering',
         'R function' = 'kernlab::spectral',
         'parameters' = 'k=13')
clust.norm.combat = append(clust.norm.combat, list(sc))
sc$result
```


```
                [,1]       [,2]      [,3]      [,4]
raw        0.3283582 0.01894452 0.1336634 0.1542625
TC         0.5522388 0.37150838 0.3431221 0.6268657
UQ         0.5522388 0.55223881 0.4204019 0.5522388
TMM        0.3283582 0.30167598 0.5522388 0.6268657
DESeq      0.4776119 0.62686567 0.4204019 0.5310245
PoissonSeq 0.3431221 0.57496136 0.4588745 0.4776119
gbnorm     0.3867244 0.55223881 0.4776119 0.6522411
                 [,5]       [,6]      [,7]      [,8]
raw        0.09217877 0.09217877 0.1702742 0.1702742
TC         0.65224111 0.55223881 0.4588745 0.5310245
UQ         0.55223881 0.55223881 0.4776119 0.4588745
TMM        0.55223881 0.55223881 0.4588745 0.5522388
DESeq      0.55223881 0.34312210 0.4204019 0.4204019
PoissonSeq 0.47761194 0.37150838 0.4776119 0.4776119
gbnorm     0.55223881 0.40298507 0.3867244 0.5310245
                [,9]      [,10]     [,11]     [,12]
raw        0.1702742 0.08660352 0.1620112 0.1791045
TC         0.4776119 0.34312210 0.5522388 0.4029851
UQ         0.4776119 0.47761194 0.5522388 0.3715084
TMM        0.4204019 0.42040185 0.5522388 0.4204019
DESeq      0.4588745 0.32835821 0.5522388 0.5522388
PoissonSeq 0.4204019 0.45887446 0.4204019 0.5522388
gbnorm     0.3867244 0.40298507 0.3867244 0.4776119
               [,13]     [,14]      [,15]     [,16]
raw        0.3283582 0.2537313 0.09217877 0.1702742
TC         0.4204019 0.4029851 0.42040185 0.5522388
UQ         0.4204019 0.4776119 0.47761194 0.3431221
TMM        0.4204019 0.5522388 0.24242424 0.5522388
DESeq      0.5522388 0.6268657 0.40298507 0.5522388
PoissonSeq 0.4776119 0.5522388 0.53102453 0.4776119
gbnorm     0.5522388 0.4588745 0.38672439 0.3867244
               [,17]     [,18]     [,19]     [,20]
raw        0.2424242 0.1791045 0.1791045 0.1702742
TC         0.5310245 0.5522388 0.4776119 0.5749614
UQ         0.5522388 0.5310245 0.5522388 0.3145743
TMM        0.3283582 0.3016760 0.5522388 0.4204019
DESeq      0.4204019 0.6268657 0.5522388 0.5522388
PoissonSeq 0.4776119 0.4204019 0.4776119 0.5310245
gbnorm     0.4776119 0.4776119 0.5522388 0.4776119
```

#### Similarity with tissue-based clusters

Similarity with the tissue-based clusters is measured by adjusted rand index.


```
cl_summary.df = lapply(clust.norm.combat, function(x) {
    str(x)
    data.frame('Algorithm' = x$algo,
               'R function' = x$`R function`,
               'Clustering parameters' = x$parameters, check.names = FALSE) %>%
        cbind(data.frame(t(x$result)))
}) %>%
    do.call(rbind, .)
```


```
List of 4
 $ result    : Named num [1:7] 0.0764 0.3193 0.3017 0.3017 0.3017 ...
  ..- attr(*, "names")= chr [1:7] "raw" "TC" "UQ" "TMM" ...
 $ algo      : chr "Hierarchical clustering"
 $ R function: chr "hclust"
 $ parameters: chr "method=complete;dist=euclidean"
List of 4
 $ result    : Named num [1:7] 0 0.308 0.308 0.308 0.308 ...
  ..- attr(*, "names")= chr [1:7] "raw" "TC" "UQ" "TMM" ...
 $ algo      : chr "Hierarchical clustering"
 $ R function: chr "hclust"
 $ parameters: chr "method=average;dist=euclidean"
List of 4
 $ result    : Named num [1:7] 0.00228 0.24789 0.30168 0.30168 0.30168 ...
  ..- attr(*, "names")= chr [1:7] "raw" "TC" "UQ" "TMM" ...
 $ algo      : chr "Hierarchical clustering"
 $ R function: chr "hclust"
 $ parameters: chr "method=complete;dist=manhanttan"
List of 4
 $ result    : Named num [1:7] 0.00468 0.18033 0.25743 0.22222 0.22222 ...
  ..- attr(*, "names")= chr [1:7] "raw" "TC" "UQ" "TMM" ...
 $ algo      : chr "Hierarchical clustering"
 $ R function: chr "hclust"
 $ parameters: chr "method=average;dist=manhanttan"
List of 4
 $ result    : Named num [1:7] 0.111 0.222 0.222 0.222 0.222 ...
  ..- attr(*, "names")= chr [1:7] "raw" "TC" "UQ" "TMM" ...
 $ algo      : chr "Hierarchical clustering"
 $ R function: chr "hclust"
 $ parameters: chr "method=single;dist=1-cor"
List of 4
 $ result    : num [1:7, 1:20] 0.162 0.425 0.357 0.357 0.344 ...
  ..- attr(*, "dimnames")=List of 2
  .. ..$ : chr [1:7] "raw" "TC" "UQ" "TMM" ...
  .. ..$ : NULL
 $ algo      : chr "K-means"
 $ R function: chr "stats::kmeans"
 $ parameters: chr "method=Hatigan-Wong; k = 13"
List of 4
 $ result    : num [1:7, 1:20] 0.328 0.552 0.552 0.328 0.478 ...
  ..- attr(*, "dimnames")=List of 2
  .. ..$ : chr [1:7] "raw" "TC" "UQ" "TMM" ...
  .. ..$ : NULL
 $ algo      : chr "Spectral clustering"
 $ R function: chr "kernlab::spectral"
 $ parameters: chr "k=13"
```


```
cl_summary.df
```


Rank correlation between clustering methods


```
cor.rank = cl_summary.df[,names(norm.combat.stanford)] %>%
    t() %>%
    cor(method = 'spearman')
pheatmap::pheatmap(1 - cor.rank,
                   cellwidth = 10,
                   cellheight = 10,
                   labels_row = cl_summary.df$Algorithm,
                   labels_col = cl_summary.df$Algorithm)
```


Differently normalized counts resulted in different clustering patterns. Each configuration of normalization methods


```
cl_summary.df[,names(norm.combat.stanford)] %>%
    apply(MARGIN = 1, FUN = rank) %>%
    t() %>%
    summary()
```


```
      raw              TC            UQ       
 Min.   :1.000   Min.   :2.0   Min.   :2.000  
 1st Qu.:1.000   1st Qu.:3.0   1st Qu.:3.500  
 Median :1.000   Median :5.0   Median :4.500  
 Mean   :1.078   Mean   :4.7   Mean   :4.744  
 3rd Qu.:1.000   3rd Qu.:6.0   3rd Qu.:6.000  
 Max.   :2.000   Max.   :7.0   Max.   :7.000  
      TMM            DESeq         PoissonSeq   
 Min.   :1.000   Min.   :2.000   Min.   :1.000  
 1st Qu.:3.000   1st Qu.:3.500   1st Qu.:3.000  
 Median :4.500   Median :4.000   Median :4.500  
 Mean   :4.378   Mean   :4.511   Mean   :4.456  
 3rd Qu.:5.500   3rd Qu.:5.500   3rd Qu.:6.000  
 Max.   :7.000   Max.   :7.000   Max.   :7.000  
     gbnorm     
 Min.   :1.000  
 1st Qu.:3.000  
 Median :4.500  
 Mean   :4.133  
 3rd Qu.:5.000  
 Max.   :7.000
```


```
sessionInfo()
```


```
R version 3.5.3 (2019-03-11)
Platform: x86_64-pc-linux-gnu (64-bit)
Running under: Ubuntu 16.04.1 LTS

Matrix products: default
BLAS: /usr/lib/libblas/libblas.so.3.6.0
LAPACK: /usr/lib/lapack/liblapack.so.3.6.0

locale:
 [1] LC_CTYPE=en_US.UTF-8       LC_NUMERIC=C              
 [3] LC_TIME=en_US.UTF-8        LC_COLLATE=en_US.UTF-8    
 [5] LC_MONETARY=en_US.UTF-8    LC_MESSAGES=en_US.UTF-8   
 [7] LC_PAPER=en_US.UTF-8       LC_NAME=C                 
 [9] LC_ADDRESS=C               LC_TELEPHONE=C            
[11] LC_MEASUREMENT=en_US.UTF-8 LC_IDENTIFICATION=C       

attached base packages:
[1] parallel  stats     graphics  grDevices utils    
[6] datasets  methods   base     

other attached packages:
[1] ggplot2_3.1.1 magrittr_1.5 

loaded via a namespace (and not attached):
  [1] colorspace_1.4-1           
  [2] hwriter_1.3.2              
  [3] mclust_5.4.3               
  [4] dynamicTreeCut_1.63-1      
  [5] htmlTable_1.13.1           
  [6] XVector_0.22.0             
  [7] GenomicRanges_1.34.0       
  [8] base64enc_0.1-3            
  [9] rstudioapi_0.10            
 [10] bit64_0.9-7                
 [11] AnnotationDbi_1.44.0       
 [12] splines_3.5.3              
 [13] R.methodsS3_1.7.1          
 [14] DESeq_1.34.1               
 [15] geneplotter_1.60.0         
 [16] knitr_1.22                 
 [17] PoissonSeq_1.1.2           
 [18] Formula_1.2-3              
 [19] apcluster_1.4.7            
 [20] TCC_1.22.1                 
 [21] Rsamtools_1.34.1           
 [22] packrat_0.5.0              
 [23] annotate_1.60.1            
 [24] baySeq_2.16.0              
 [25] kernlab_0.9-27             
 [26] cluster_2.0.8              
 [27] R.oo_1.22.0                
 [28] pheatmap_1.0.12            
 [29] compiler_3.5.3             
 [30] httr_1.4.0                 
 [31] backports_1.1.4            
 [32] assertthat_0.2.1           
 [33] Matrix_1.2-17              
 [34] lazyeval_0.2.2             
 [35] limma_3.38.3               
 [36] acepack_1.4.1              
 [37] htmltools_0.3.6            
 [38] prettyunits_1.0.2          
 [39] tools_3.5.3                
 [40] igraph_1.2.4               
 [41] gtable_0.3.0               
 [42] glue_1.3.1                 
 [43] GenomeInfoDbData_1.2.0     
 [44] dplyr_0.8.0.1              
 [45] ShortRead_1.40.0           
 [46] Rcpp_1.0.2                 
 [47] Biobase_2.42.0             
 [48] Biostrings_2.50.2          
 [49] nlme_3.1-139               
 [50] rtracklayer_1.42.2         
 [51] xfun_0.6                   
 [52] stringr_1.4.0              
 [53] ROC_1.58.0                 
 [54] XML_3.98-1.19              
 [55] edgeR_3.24.3               
 [56] zlibbioc_1.28.0            
 [57] MASS_7.3-51.3              
 [58] scales_1.0.0               
 [59] aroma.light_3.12.0         
 [60] hms_0.4.2                  
 [61] SummarizedExperiment_1.12.0
 [62] gbnorm_0.0.0.9000          
 [63] RColorBrewer_1.1-2         
 [64] yaml_2.2.0                 
 [65] memoise_1.1.0              
 [66] RUVSeq_1.16.1              
 [67] gridExtra_2.3              
 [68] biomaRt_2.38.0             
 [69] rpart_4.1-15               
 [70] latticeExtra_0.6-28        
 [71] stringi_1.4.3              
 [72] RSQLite_2.1.1              
 [73] genefilter_1.64.0          
 [74] S4Vectors_0.20.1           
 [75] checkmate_1.9.1            
 [76] GenomicFeatures_1.34.8     
 [77] BiocGenerics_0.28.0        
 [78] BiocParallel_1.16.6        
 [79] truncnorm_1.0-8            
 [80] GenomeInfoDb_1.18.2        
 [81] rlang_0.4.0                
 [82] pkgconfig_2.0.2            
 [83] matrixStats_0.54.0         
 [84] bitops_1.0-6               
 [85] evaluate_0.13              
 [86] lattice_0.20-38            
 [87] purrr_0.3.2                
 [88] labeling_0.3               
 [89] GenomicAlignments_1.18.1   
 [90] htmlwidgets_1.3            
 [91] bit_1.1-14                 
 [92] tidyselect_0.2.5           
 [93] plyr_1.8.4                 
 [94] DESeq2_1.22.2              
 [95] R6_2.4.0                   
 [96] IRanges_2.16.0             
 [97] Hmisc_4.2-0                
 [98] DelayedArray_0.8.0         
 [99] DBI_1.0.0                  
[100] withr_2.1.2                
[101] pillar_1.3.1               
[102] foreign_0.8-71             
[103] mgcv_1.8-28                
[104] survival_2.44-1.1          
[105] abind_1.4-5                
[106] RCurl_1.95-4.12            
[107] nnet_7.3-12                
[108] tibble_2.1.1               
[109] EDASeq_2.16.3              
[110] crayon_1.3.4               
[111] rmarkdown_1.12             
[112] progress_1.2.0             
[113] locfit_1.5-9.1             
[114] grid_3.5.3                 
[115] sva_3.31.0                 
[116] data.table_1.12.2          
[117] blob_1.1.1                 
[118] digest_0.6.18              
[119] xtable_1.8-3               
[120] dbscan_1.1-3               
[121] R.utils_2.8.0              
[122] stats4_3.5.3               
[123] munsell_0.5.0
```

## References

Anders, Simon, and Wolfgang Huber. 2010. “Differential Expression Analysis for Sequence Count Data.” *Genome Biology* 11: R106. https://doi.org/10.1186/gb-2010-11-10-r106.

Bullard, James H., Elizabeth Purdom, Kasper D. Hansen, and Sandrine Dudoit. 2010. “Evaluation of Statistical Methods for Normalization and Differential Expression in mRNA-Seq Experiments.” *BMC Bioinformatics* 11: 94. https://doi.org/10.1186/1471-2105-11-94.

Chan, Esther T., Gerald T. Quon, Gordon Chua, Tomas Babak, Miles Trochesset, Ralph A. Zirngibl, Jane Aubin, et al. 2009. “Conservation of Core Gene Expression in Vertebrate Tissues.” *Journal of Biology* 8 (3): 33. https://doi.org/10.1186/jbiol130.

Gilad, Yoav, and Orna Mizrahi-Man. 2015. “A Reanalysis of Mouse ENCODE Comparative Gene Expression Data.” *F1000Research* 4 (May): 121. https://doi.org/10.12688/f1000research.6536.1.

Johnson, W. Evan, Cheng Li, and Ariel Rabinovic. 2007. “Adjusting Batch Effects in Microarray Expression Data Using Empirical Bayes Methods.” *Biostatistics* 8 (1): 118–27. https://doi.org/10.1093/biostatistics/kxj037.

Leek, Jeffrey T., W. Evan Johnson, Hilary S. Parker, Andrew E. Jaffe, and John D. Storey. 2012. “The Sva Package for Removing Batch Effects and Other Unwanted Variation in High-Throughput Experiments.” *Bioinformatics* 28 (6): 882–83. https://doi.org/10.1093/bioinformatics/bts034.

Li, Jun, Daniela M. Witten, Iain M. Johnstone, and Robert Tibshirani. 2012. “Normalization, Testing, and False Discovery Rate Estimation for RNA-Sequencing Data.” *Biostatistics* 13 (3): 523–38. https://doi.org/10.1093/biostatistics/kxr031.

Mizrahi-Man, Orna, and Yoav Gilad. 2015. “Data Files and Codes Used in the Reanalysis of the Mouse Encode Comparative Gene Expression Data.” Zenodo. https://doi.org/10.5281/zenodo.17606.

Robinson, Mark D., and Alicia Oshlack. 2010. “A Scaling Normalization Method for Differential Expression Analysis of RNA-Seq Data.” *Genome Biology* 11: R25. https://doi.org/10.1186/gb-2010-11-3-r25.

Sudmant, Peter H., Maria S. Alexis, and Christopher B. Burge. 2015. “Meta-Analysis of RNA-Seq Expression Data Across Species, Tissues and Studies.” *Genome Biology* 16: 287. https://doi.org/10.1186/s13059-015-0853-4.

LS0tCnRpdGxlOiAiRWZmZWN0IG9mIG5vcm1hbGl6YXRpb24gb24gYmF0Y2ggZWZmZWN0IGNvcnJlY3Rpb24iCm91dHB1dDoKICAgIGh0bWxfbm90ZWJvb2s6CiAgICAgICAgdG9jOiB5ZXMKICAgICAgICB0b2NfZGVwdGg6IDUKYmlibGlvZ3JhcGh5OiBnYm5vcm0uYmliCm5vY2l0ZTogfCAKICBASm9obnNvbjoyMDA3OkFkanVzdGluZywgQExlZWs6MjAxMjpzdmEKLS0tCgpgYGB7ciBzZXR1cCwgaW5jbHVkZT1GQUxTRX0Ka25pdHI6Om9wdHNfY2h1bmskc2V0KGVjaG8gPSBUUlVFKQpvcHRpb25zKHdpZHRoID0gMTIwLCBzdHJpbmdzQXNGYWN0b3JzID0gRkFMU0UpCnNvdXJjZSgnLi4vUi91dGlscy5SJykKc291cmNlKCcuLi9SL3Bsb3QuUicpCnNvdXJjZSgnLi4vUi9wcm9qZWN0X3BjYS5SJykKCmxpYnJhcnkobWFncml0dHIpCmxpYnJhcnkoZ2dwbG90MikKbGlicmFyeShwYXJhbGxlbCkKCiMgZ2VuZXMgaW4gY29sdW1ucwpyZW1vdmVfaW52YXJpYW50X2dlbmVzIDwtIGZ1bmN0aW9uKFgpIHsKICAgIGNvbHZhciA9IGFwcGx5KFgsIE1BUkdJTiA9IDIsIEZVTiA9IHZhcikKICAgIHJldHVybihYWywtd2hpY2goY29sdmFyID09IDApXSkKfQoKcmVtb3ZlX2xvd2VzdF9xdWFudGlsZSA8LSBmdW5jdGlvbihYLCBwID0gMC4zKSB7CiAgICBjb2xzdW0gPSBjb2xTdW1zKFgpCiAgICByZXR1cm4oWFssd2hpY2goY29sc3VtID49IHF1YW50aWxlKGNvbHN1bSwgcCkpXSkKfQoKcGxvdF9wY2EgPC0gZnVuY3Rpb24oZGYsIHNoYXBlLCBjb2xvcikgewogICAgcCA9IGdncGxvdChkZikgKwogICAgICAgIGdlb21fcG9pbnQoYWVzX3N0cmluZyh4PSdQQzEnLHk9J1BDMicsIHNoYXBlID0gc2hhcGUsIGNvbG9yID0gY29sb3IpKSArCiAgICAgICAgc2NhbGVfc2hhcGVfbWFudWFsKHZhbHVlcyA9IGMoMCwxLDIsMyw0LDUsNiw3LDgsOSwxMCwxMiwxMykpCiAgICByZXR1cm4ocCkKfQpgYGAKCiMjIFByZXBhcmUgZGF0YSBzZXQKClRoZSBkYXRhIHdlcmUgcHJvdmlkZWQgYXMgY29tcGFuaW9uIHRvIHRoZSByZS1hbmFseXNpcyBieSBHaWxhZCBhbmQgTWl6cmFoaS1NYW4gW0BHaWxhZDoyMDE1OnJlYW5hbHlzaXNdLCBhdCBbZG9pOjEwLjUyODEvemVub2RvLjE3NjA2XShodHRwOi8vZG9pLm9yZy8xMC41MjgxL3plbm9kby4xNzYwNikgW0BNaXpyYWhpLU1hbjoyMDE1OkRhdGFdLgoKYGBge3J9CmdlbmVEZXRhaWxzID0gZGF0YS50YWJsZTo6ZnJlYWQoJy4uL2RhdGEvR2lsYWQyMDE1L1JfaW5wdXRfZmlsZXMvb3J0aG9sb2dfR0NfdGFibGUudHh0JykKc3RhbmZvcmQubWV0YSA9IGRhdGEudGFibGU6OmZyZWFkKCcuLi9kYXRhL0dpbGFkMjAxNS9SX2lucHV0X2ZpbGVzL1N0YW5mb3JkX2RhdGFzZXRzLnR4dCcsaGVhZGVyPUZBTFNFKSAlPiUKICAgIHNldF9uYW1lcyhjKCdsYWJlbCcsICdzZXF1ZW5jZV9pZGVudGlmaWVyJywgJ3NwZWNpZXMnLCAndGlzc3VlJykpCnN0YW5mb3JkLmZwa20gPSBkYXRhLnRhYmxlOjpmcmVhZCgnLi4vZGF0YS9HaWxhZDIwMTUvUl9pbnB1dF9maWxlcy9TdGFuZm9yZF9kYXRhc2V0c19mcGttTWF0LnR4dCcpICU+JQogICAgdCgpICU+JQogICAgc2V0X2NvbG5hbWVzKGdlbmVEZXRhaWxzJGh1bWFuX25hbWUpICU+JQogICAgc2V0X3Jvd25hbWVzKHN0YW5mb3JkLm1ldGEkbGFiZWwpICU+JQogICAgc3Vic2V0KC4sIFRSVUUsICFncmVwbCgnbXQtJywgeCA9IGdlbmVEZXRhaWxzJG1vdXNlX25hbWUpKSAlPiUKICAgIHJlbW92ZV9pbnZhcmlhbnRfZ2VuZXMoKQpzdGFuZm9yZC5jbnQgPSBkYXRhLnRhYmxlOjpmcmVhZCgnLi4vZGF0YS9HaWxhZDIwMTUvUl9pbnB1dF9maWxlcy9TdGFuZm9yZF9kYXRhc2V0c19yYXdDb3VudHNNYXQudHh0JykgJT4lCiAgICB0KCkgJT4lCiAgICBzZXRfY29sbmFtZXMoZ2VuZURldGFpbHMkaHVtYW5fbmFtZSkgJT4lCiAgICBzZXRfcm93bmFtZXMoc3RhbmZvcmQubWV0YSRsYWJlbCkgJT4lCiAgICBzdWJzZXQoLiwgVFJVRSwgIWdyZXBsKCdtdC0nLCB4ID0gZ2VuZURldGFpbHMkbW91c2VfbmFtZSkpICU+JQogICAgcmVtb3ZlX2ludmFyaWFudF9nZW5lcygpIApzdGFuZm9yZC5jbnQgJT4lCiAgICByZW1vdmVfbG93ZXN0X3F1YW50aWxlKHAgPSAwLjMpICU+JQogICAgcHJvamVjdC5wY2Eoc2VsZWN0ZWQucGMgPSBjKDEsMikpICU+JQogICAgY2JpbmQoc3RhbmZvcmQubWV0YSkgJT4lCiAgICBwbG90X3BjYShzaGFwZSA9ICd0aXNzdWUnLCBjb2xvciA9ICdzcGVjaWVzJykKc3RhbmZvcmQuY250ICU+JQogICAgcmVtb3ZlX2xvd2VzdF9xdWFudGlsZShwID0gMC4zKSAlPiUKICAgIHByb2plY3QucGNhKHNlbGVjdGVkLnBjID0gYygxLDIpLCBzY2FsZSA9IFRSVUUpICU+JQogICAgY2JpbmQoc3RhbmZvcmQubWV0YSkgJT4lCiAgICBwbG90X3BjYShzaGFwZSA9ICd0aXNzdWUnLCBjb2xvciA9ICdzcGVjaWVzJykKYGBgCgojIyBOb3JtYWxpemF0aW9uIG1ldGhvZHMKClRoZSBmb2xsb3dpbmcgbm9ybWFsaXphdGlvbiBwcm9jZWR1cmVzIGFyZSBleGFtaW5lZCBpbiB0aGlzIGFuYWx5c2lzOgoKKiBObyBzY2FsaW5nIChyYXcpCiogVG90YWwgQ291bnQgKFRDKQoqIFVwcGVyIFF1YW50aWxlIChVUSkgW0BCdWxsYXJkOjIwMTA6RXZhbHVhdGlvbl0KKiBUTU0gW0BSb2JpbnNvbjoyMDEwOnNjYWxpbmddCiogREVTZXEgW0BBbmRlcnM6MjAxMDpEaWZmZXJlbnRpYWxdCiogUG9pc3NvblNlcSBbQExpOjIwMTI6Tm9ybWFsaXphdGlvbl0KKiBHcmFwaC1iYXNlZCBub3JtYWxpemF0aW9uIChnYm5vcm0pIChkZXNjcmliZWQgaW4gdGhpcyBtYW51c2NyaXB0KQoKYGBge3J9Cm1ldGhvZHMgPSBsaXN0KCdyYXcnID0gTkEsCiAgICAgICAgICAgICAgICdUQycgPSBOQSwKICAgICAgICAgICAgICAgJ1VRJyA9IGdibm9ybTo6bm9ybWFsaXplLmJ5LnVxLAogICAgICAgICAgICAgICAnVE1NJyA9IGdibm9ybTo6bm9ybWFsaXplLmJ5LnRtbSwKICAgICAgICAgICAgICAgJ0RFU2VxJyA9IGdibm9ybTo6bm9ybWFsaXplLmJ5LmRlc2VxLAogICAgICAgICAgICAgICAnUG9pc3NvblNlcScgPSBnYm5vcm06Om5vcm1hbGl6ZS5ieS5wb2lzc29uc2VxLAogICAgICAgICAgICAgICAnZ2Jub3JtJyA9IE5BKQptZXRob2RzJHJhdyA8LSBmdW5jdGlvbihYKSB7CiAgICByZXR1cm4oWCkKfQoKCm1ldGhvZHMkVEMgPC0gZnVuY3Rpb24oWCkgewogICAgc2NhbGVGYWN0b3JzID0gYXBwbHkoWCwgMSwgc3VtKQogICAgc2NhbGVGYWN0b3JzID0gc2NhbGVGYWN0b3JzIC8gZ2VvbS5tZWFuKHNjYWxlRmFjdG9ycykKICAgIHJldHVybihzd2VlcChYLCAxLCBTVEFUUyA9IHNjYWxlRmFjdG9ycywgRlVOID0gIi8iKSkKfQoKbWV0aG9kcyRnYm5vcm0gPC0gZnVuY3Rpb24oWCkgewogICAgcmVmcyA9IGdibm9ybTo6Z2V0LnJlZmVyZW5jZXMuYXBjbHVzdGVyKFgsCiAgICAgICAgICAgICAgICAgICAgICAgICAgICAgICAgICAgY29yLm1ldGhvZCA9ICdwZWFyc29uJywKICAgICAgICAgICAgICAgICAgICAgICAgICAgICAgICAgICBtaW4uY291bnQgPSAxMCkgJT4lCiAgICAgICAgYCRgKCdOYW1lJykKICAgIFgubm9ybWVkID0gd2hpY2goY29sbmFtZXMoWCkgJWluJSByZWZzKSAlPiUKICAgICAgICBnYm5vcm06Om5vcm1hbGl6ZS5ieS5yZWZzKFgsIC4sIHNjYWxlID0gVFJVRSkKICAgIHJldHVybihYLm5vcm1lZCkKfQpgYGAKCgpgYGB7cixldmFsPUZBTFNFfQpub3JtLnN0YW5mb3JkIDwtIG1jbGFwcGx5KDE6bGVuZ3RoKG1ldGhvZHMpLCBtYy5jb3JlcyA9IG1pbig0LCBsZW5ndGgobWV0aG9kcyksIGRldGVjdENvcmVzKCkgLSAxKSwgRlVOID0gZnVuY3Rpb24oaSkgewogICAgc3RhcnQgPSBwcm9jLnRpbWUoKQogICAgWC5ub3JtZWQgPC0gZG8uY2FsbChtZXRob2RzW1tpXV0sIGxpc3Qoc3RhbmZvcmQuY250KSkKICAgIGR0ID0gKHByb2MudGltZSgpIC0gc3RhcnQpWydlbGFwc2VkJ10KICAgIHJldHVybihsaXN0KCdtZXRob2QnID0gbmFtZXMobWV0aG9kcylbW2ldXSwKICAgICAgICAgICAgICAgICd0aW1lJyA9IGR0LAogICAgICAgICAgICAgICAgJ25vcm1hbGl6ZWRDb3VudHMnID0gWC5ub3JtZWQpKQp9KSAlPiUKICAgIHNldF9uYW1lcyhuYW1lcyhtZXRob2RzKSkKYGBgCgpgYGB7cixldmFsPUZBTFNFfQpzYXZlUkRTKG5vcm0uc3RhbmZvcmQsIGZpbGUgPSAnc3RhbmZvcmQtbm9ybWFsaXplZC5SRFMnKQpgYGAKYGBge3J9CiMgUHJlLWNvbXB1dGVkIG5vcm1hbGl6YXRpb25zIGFyZSBsb2FkZWQgdG8gc2F2ZSBydW5uaW5nIHRpbWUsIGluIGNhc2UgdGhleSBhcmUgYXZhaWxhYmxlCm5vcm0uc3RhbmZvcmQgPSByZWFkUkRTKCdzdGFuZm9yZC1ub3JtYWxpemVkLlJEUycpCmBgYAoKIyMjIFBhaXJ3aXNlIGNvbmRpdGlvbi1udW1iZXItYmFzZWQgZGV2aWF0aW9ucwoKJGNkZXYkIGJldHdlZW4gZXZlcnkgcGFpciBvZiBleHByZXNzaW9uIG1hdHJpY2VzIGlzIGNhbGN1bGF0ZWQgYmVsb3cuIEFsbCBub3JtYWxpemVkIGNvdW50IG1hdHJpY2VzIGFyZSBjbG9zZSB0byBvbmUgYW5vdGhlciBidXQgZGlmZmVyIHNpZ25pZmljYW50bHkgZnJvbSB0aGUgcmF3IGNvdW50IG1hdHJpeC4KCmBgYHtyfQptY2xhcHBseShuYW1lcyhub3JtLnN0YW5mb3JkKSwgbWMuY29yZXMgPSBtaW4obGVuZ3RoKG5vcm0uc3RhbmZvcmQpLCBkZXRlY3RDb3JlcygpIC0gMiksIEZVTiA9IGZ1bmN0aW9uKGkpIHsKICAgIHNhcHBseShuYW1lcyhub3JtLnN0YW5mb3JkKSwgZnVuY3Rpb24oaikgewogICAgICAgIGdibm9ybTo6Y2Rldihub3JtLnN0YW5mb3JkW1tpXV0kbm9ybWFsaXplZENvdW50cywgbm9ybS5zdGFuZm9yZFtbal1dJG5vcm1hbGl6ZWRDb3VudHMpICU+JQogICAgICAgICAgICByZXR1cm4oKQogICAgfSkKfSkgJT4lCiAgICBkby5jYWxsKHJiaW5kLCAuKSAlPiUKICAgIHNldF9yb3duYW1lcyhuYW1lcyhub3JtLnN0YW5mb3JkKSkgJT4lCiAgICBzZXRfY29sbmFtZXMobmFtZXMobm9ybS5zdGFuZm9yZCkpCmBgYAoKIyMgUENBCgojIyMgQmVmb3JlIGJhdGNoIGNvcnJlY3Rpb24KCmBgYHtyfQpmb3IgKGkgaW4gbmFtZXMobm9ybS5zdGFuZm9yZCkpIHsKICAgIHggPSBub3JtLnN0YW5mb3JkW1tpXV0kbm9ybWFsaXplZENvdW50cwogICAgcCA9IHJlbW92ZV9sb3dlc3RfcXVhbnRpbGUoeCwgcCA9IDAuMykgJT4lCiAgICAgICAgcHJvamVjdC5wY2Eoc2VsZWN0ZWQucGMgPSBjKDEsMiksY2VudGVyID0gVFJVRSwgc2NhbGUgPSBUUlVFKSAlPiUKICAgICAgICBjYmluZChzdGFuZm9yZC5tZXRhKSAlPiUKICAgICAgICBwbG90X3BjYShzaGFwZSA9ICd0aXNzdWUnLCBjb2xvciA9ICdzcGVjaWVzJykgICArCiAgICAgICAgZ3VpZGVzKHNoYXBlPWd1aWRlX2xlZ2VuZChuY29sID0gMikpICsKICAgICAgICBnZ3RpdGxlKGkpCiAgICBwcmludChwKQp9IAoKYGBgCgpgYGB7cn0KZm9yIChpIGluIG5hbWVzKG5vcm0uc3RhbmZvcmQpKSB7CiAgICB4ID0gbm9ybS5zdGFuZm9yZFtbaV1dJG5vcm1hbGl6ZWRDb3VudHMKICAgIHAgPSByZW1vdmVfbG93ZXN0X3F1YW50aWxlKHgsIHAgPSAwLjMpICU+JQogICAgICAgIHByb2plY3QucGNhKHNlbGVjdGVkLnBjID0gYygxLDIpLGNlbnRlciA9IFRSVUUsIHNjYWxlID0gVFJVRSkgJT4lCiAgICAgICAgY2JpbmQoc3RhbmZvcmQubWV0YSkgJT4lCiAgICAgICAgcGxvdF9wY2Eoc2hhcGUgPSAnc2VxdWVuY2VfaWRlbnRpZmllcicsIGNvbG9yID0gJ3NwZWNpZXMnKSAgICsKICAgICAgICBnZ3RpdGxlKGkpCiAgICBwcmludChwKQp9IAoKYGBgCgojIyMgQWZ0ZXIgYmF0Y2ggY29ycmVjdGlvbiB3aXRoIGNvbWJhdAoKYGBge3J9Cm5vcm0uY29tYmF0LnN0YW5mb3JkID0gbGFwcGx5KG5hbWVzKG5vcm0uc3RhbmZvcmQpLCBmdW5jdGlvbihpKSB7CiAgICB4ID0gbm9ybS5zdGFuZm9yZFtbaV1dJG5vcm1hbGl6ZWRDb3VudHMgJT4lCiAgICAgICAgYWRkKDEpICU+JQogICAgICAgIGxvZzIoKSAlPiUKICAgICAgICB0KCkgJT4lCiAgICAgICAgc3ZhOjpDb21CYXQoYmF0Y2ggPSBzdGFuZm9yZC5tZXRhJHNlcXVlbmNlX2lkZW50aWZpZXIsCiAgICAgICAgICAgICAgICAgICAgbW9kID0gbW9kZWwubWF0cml4KH4xLCBkYXRhID0gZGF0YS5mcmFtZSh0aXNzdWUgPSBzdGFuZm9yZC5tZXRhJHRpc3N1ZSwgc3BlY2llcyA9IHN0YW5mb3JkLm1ldGEkc3BlY2llcykpLAogICAgICAgICAgICAgICAgICAgIHBhci5wcmlvciA9IFRSVUUpICU+JQogICAgICAgIHQoKQogICAgcmV0dXJuKHgpCn0pICU+JQogICAgc2V0X25hbWVzKG5hbWVzKG5vcm0uc3RhbmZvcmQpKQpgYGAKYGBge3J9CmZvciAoaSBpbiBuYW1lcyhub3JtLmNvbWJhdC5zdGFuZm9yZCkpIHsKICAgIHggPSBub3JtLmNvbWJhdC5zdGFuZm9yZFtbaV1dCiAgICBwID0gcmVtb3ZlX2xvd2VzdF9xdWFudGlsZSh4LCBwID0gMC4zKSAlPiUKICAgICAgICBwcm9qZWN0LnBjYShzZWxlY3RlZC5wYyA9IGMoMSwyKSxjZW50ZXIgPSBUUlVFLCBzY2FsZSA9IFRSVUUpICU+JQogICAgICAgIGNiaW5kKHN0YW5mb3JkLm1ldGEpICU+JQogICAgICAgIHBsb3RfcGNhKHNoYXBlID0gJ3Rpc3N1ZScsIGNvbG9yID0gJ3NwZWNpZXMnKSAgICsKICAgICAgICBnZ3RpdGxlKGkpCiAgICBwcmludChwKQp9IAoKYGBgCgpgYGB7cn0KZm9yIChpIGluIG5hbWVzKG5vcm0uY29tYmF0LnN0YW5mb3JkKSkgewogICAgeCA9IG5vcm0uY29tYmF0LnN0YW5mb3JkW1tpXV0KICAgIHAgPSByZW1vdmVfbG93ZXN0X3F1YW50aWxlKHgsIHAgPSAwLjMpICU+JQogICAgICAgIHByb2plY3QucGNhKHNlbGVjdGVkLnBjID0gYygxLDIpLGNlbnRlciA9IFRSVUUsIHNjYWxlID0gVFJVRSkgJT4lCiAgICAgICAgY2JpbmQoc3RhbmZvcmQubWV0YSkgJT4lCiAgICAgICAgcGxvdF9wY2Eoc2hhcGUgPSAnc3BlY2llcycsIGNvbG9yID0gJ3NlcXVlbmNlX2lkZW50aWZpZXInKSAgICsKICAgICAgICBnZ3RpdGxlKGkpCiAgICBwcmludChwKQp9IAoKYGBgCgojIyBTYW1wbGUgY2x1c3RlcmluZwoKIyMjIEJlZm9yZSBiYXRjaCBjb3JyZWN0aW9uCgpgYGB7cn0KCmhjbHVzdC5ldWNsaWQuc3RhbmZvcmQgPSBsYXBwbHkobmFtZXMobm9ybS5zdGFuZm9yZCksIGZ1bmN0aW9uKGkpIHsKICAgIHggPSBub3JtLnN0YW5mb3JkW1tpXV0kbm9ybWFsaXplZENvdW50cwogICAgcmVtb3ZlX2xvd2VzdF9xdWFudGlsZSh4KSAlPiUKICAgICAgICBkaXN0KG1ldGhvZCA9ICdldWNsaWRlYW4nKSAlPiUKICAgICAgICBoY2x1c3QoKQp9KSAlPiUKICAgIHNldF9uYW1lcyhuYW1lcyhub3JtLnN0YW5mb3JkKSkKc2FwcGx5KGhjbHVzdC5ldWNsaWQuc3RhbmZvcmQsIGZ1bmN0aW9uKHgpIHsKICAgIGN1dHJlZSh4LCBrID0gMTMpICU+JQogICAgbWNsdXN0OjphZGp1c3RlZFJhbmRJbmRleChzdGFuZm9yZC5tZXRhJHRpc3N1ZSkKfSkgJT4lCiAgICBzZXRfbmFtZXMobmFtZXMoaGNsdXN0LmV1Y2xpZC5zdGFuZm9yZCkpCmBgYApgYGB7cn0KbGFwcGx5KG5hbWVzKG5vcm0uc3RhbmZvcmQpLCBmdW5jdGlvbihpKSB7CiAgICB4ID0gbm9ybS5zdGFuZm9yZFtbaV1dJG5vcm1hbGl6ZWRDb3VudHMKICAgIHJlbW92ZV9sb3dlc3RfcXVhbnRpbGUoeCkgJT4lCiAgICAgICAgZGlzdChtZXRob2QgPSAnZXVjbGlkZWFuJykgJT4lCiAgICAgICAgaGNsdXN0KG1ldGhvZCA9ICdhdmVyYWdlJykKfSkgJT4lCiAgICBzZXRfbmFtZXMobmFtZXMobm9ybS5zdGFuZm9yZCkpICU+JQogICAgc2FwcGx5KGZ1bmN0aW9uKHgpIHsKICAgIGN1dHJlZSh4LCBrID0gMTMpICU+JQogICAgbWNsdXN0OjphZGp1c3RlZFJhbmRJbmRleChzdGFuZm9yZC5tZXRhJHRpc3N1ZSkKfSkgJT4lCiAgICBzZXRfbmFtZXMobmFtZXMobm9ybS5zdGFuZm9yZCkpCmBgYAoKYGBge3J9CmxhcHBseShuYW1lcyhub3JtLnN0YW5mb3JkKSwgZnVuY3Rpb24oaSkgewogICAgeCA9IG5vcm0uc3RhbmZvcmRbW2ldXSRub3JtYWxpemVkQ291bnRzCiAgICByZW1vdmVfbG93ZXN0X3F1YW50aWxlKHgpICU+JQogICAgICAgIGRpc3QobWV0aG9kID0gJ2V1Y2xpZGVhbicpICU+JQogICAgICAgIGhjbHVzdChtZXRob2QgPSAnYXZlcmFnZScpCn0pICU+JQogICAgc2V0X25hbWVzKG5hbWVzKG5vcm0uc3RhbmZvcmQpKSAlPiUKICAgIHNhcHBseShmdW5jdGlvbih4KSB7CiAgICBjdXRyZWUoeCwgayA9IDEzKSAlPiUKICAgIG1jbHVzdDo6YWRqdXN0ZWRSYW5kSW5kZXgoc3RhbmZvcmQubWV0YSR0aXNzdWUpCn0pICU+JQogICAgc2V0X25hbWVzKG5hbWVzKG5vcm0uc3RhbmZvcmQpKQpgYGAKYGBge3J9CmxhcHBseShuYW1lcyhub3JtLnN0YW5mb3JkKSwgZnVuY3Rpb24oaSkgewogICAgeCA9IG5vcm0uc3RhbmZvcmRbW2ldXSRub3JtYWxpemVkQ291bnRzCiAgICByZW1vdmVfbG93ZXN0X3F1YW50aWxlKHgpICU+JQogICAgICAgIGRpc3QobWV0aG9kID0gJ21hbmhhdHRhbicpICU+JQogICAgICAgIGhjbHVzdChtZXRob2QgPSAnY29tcGxldGUnKQp9KSAlPiUKICAgIHNldF9uYW1lcyhuYW1lcyhub3JtLnN0YW5mb3JkKSkgJT4lCiAgICBzYXBwbHkoZnVuY3Rpb24oeCkgewogICAgY3V0cmVlKHgsIGsgPSAxMykgJT4lCiAgICBtY2x1c3Q6OmFkanVzdGVkUmFuZEluZGV4KHN0YW5mb3JkLm1ldGEkdGlzc3VlKQp9KSAlPiUKICAgIHNldF9uYW1lcyhuYW1lcyhub3JtLnN0YW5mb3JkKSkKYGBgCgpgYGB7cn0KbGFwcGx5KG5hbWVzKG5vcm0uc3RhbmZvcmQpLCBmdW5jdGlvbihpKSB7CiAgICB4ID0gbm9ybS5zdGFuZm9yZFtbaV1dJG5vcm1hbGl6ZWRDb3VudHMKICAgIHJlbW92ZV9sb3dlc3RfcXVhbnRpbGUoeCkgJT4lCiAgICAgICAgZGlzdChtZXRob2QgPSAnbWFuaGF0dGFuJykgJT4lCiAgICAgICAgaGNsdXN0KG1ldGhvZCA9ICdhdmVyYWdlJykKfSkgJT4lCiAgICBzZXRfbmFtZXMobmFtZXMobm9ybS5zdGFuZm9yZCkpICU+JQogICAgc2FwcGx5KGZ1bmN0aW9uKHgpIHsKICAgIGN1dHJlZSh4LCBrID0gMTMpICU+JQogICAgbWNsdXN0OjphZGp1c3RlZFJhbmRJbmRleChzdGFuZm9yZC5tZXRhJHRpc3N1ZSkKfSkgJT4lCiAgICBzZXRfbmFtZXMobmFtZXMobm9ybS5zdGFuZm9yZCkpCmBgYAoKSy1tZWFucwoKYGBge3J9CmxhcHBseShuYW1lcyhub3JtLnN0YW5mb3JkKSwgZnVuY3Rpb24oaSkgewogICAgeCA9IG5vcm0uc3RhbmZvcmRbW2ldXSRub3JtYWxpemVkQ291bnRzCiAgICByZW1vdmVfbG93ZXN0X3F1YW50aWxlKHgpICU+JQogICAgICAgIGttZWFucyhjZW50ZXJzID0gMTMsIGl0ZXIubWF4ID0gMzApCn0pICU+JSAKICAgIHNldF9uYW1lcyhuYW1lcyhub3JtLnN0YW5mb3JkKSkgJT4lCiAgICBzYXBwbHkoZnVuY3Rpb24oeCkgewogICAgICAgIHgkY2x1c3RlciAlPiUKICAgICAgICBtY2x1c3Q6OmFkanVzdGVkUmFuZEluZGV4KHN0YW5mb3JkLm1ldGEkdGlzc3VlKQogICAgfSkgJT4lCiAgICBzZXRfbmFtZXMobmFtZXMobm9ybS5zdGFuZm9yZCkpCmBgYAoKIyMjIyBIZWF0bWFwIHVzaW5nIEV1Y2xpZGVhbiBkaXN0YW5jZQoKYGBge3IsZmlnLmhlaWdodD05fQpibHVlUGFsbGV0dGUgPSBSQ29sb3JCcmV3ZXI6OmJyZXdlci5wYWwoOSwgJ0JsdWVzJykKYmx1ZTI1NSA9IGNvbG9yUmFtcFBhbGV0dGUocmV2KGJsdWVQYWxsZXR0ZSkpKDI1NSkKcGFsMiA9IFJDb2xvckJyZXdlcjo6YnJld2VyLnBhbCgyLCAnQWNjZW50JylbMToyXSAlPiUKICAgIHNldF9uYW1lcyh1bmlxdWUoc3RhbmZvcmQubWV0YSRzcGVjaWVzKSkKcGFsNSA9IFJDb2xvckJyZXdlcjo6YnJld2VyLnBhbCg1LCAnQWNjZW50JykgJT4lCiAgICBzZXRfbmFtZXModW5pcXVlKHN0YW5mb3JkLm1ldGEkc2VxdWVuY2VfaWRlbnRpZmllcikpCnNwZWNpZXNfY29sb3JzID0gbGlzdCgnc3BlY2llcycgPSBwYWwyLAogICAgICAgICAgICAgICAgICAgICAgJ2JhdGNoJyA9IHBhbDUpCgpmb3IgKGkgaW4gbmFtZXMobm9ybS5zdGFuZm9yZCkpIHsKICAgIHggPSBub3JtLnN0YW5mb3JkW1tpXV0kbm9ybWFsaXplZENvdW50cwogICAgZGlzdC54ID0gcmVtb3ZlX2xvd2VzdF9xdWFudGlsZSh4LCBwID0gMC4zKSAlPiUKICAgICAgICBkaXN0KG1ldGhvZCA9ICdldWNsaWRlYW4nKQogICAgaGNsdXN0LnggPSBoY2x1c3QoZGlzdC54LCBtZXRob2QgPSAnYXZlcmFnZScpCiAgICBwaGVhdG1hcDo6cGhlYXRtYXAoYXMubWF0cml4KGRpc3QueCksCiAgICAgICAgICAgICAgICAgICAgICAgY2x1c3Rlcl9yb3dzID0gaGNsdXN0LngsIGNsdXN0ZXJfY29scyA9IGhjbHVzdC54LAogICAgICAgICAgICAgICAgICAgICAgIGxhYmVsc19yb3cgPSBzdGFuZm9yZC5tZXRhJGxhYmVsLCBsYWJlbHNfY29sID0gc3RhbmZvcmQubWV0YSRzZXF1ZW5jZV9pZGVudGlmaWVyLAogICAgICAgICAgICAgICAgICAgICAgIGFubm90YXRpb25fcm93ID0gZGF0YS5mcmFtZSgnc3BlY2llcycgPSBzdGFuZm9yZC5tZXRhJHNwZWNpZXMsIHJvdy5uYW1lcyA9IHN0YW5mb3JkLm1ldGEkbGFiZWwpLAogICAgICAgICAgICAgICAgICAgICAgIGFubm90YXRpb25fY29sID0gZGF0YS5mcmFtZSgnYmF0Y2gnID0gc3RhbmZvcmQubWV0YSRzZXF1ZW5jZV9pZGVudGlmaWVyLCByb3cubmFtZXMgPSBzdGFuZm9yZC5tZXRhJGxhYmVsKSwKICAgICAgICAgICAgICAgICAgICAgICBhbm5vdGF0aW9uX2NvbG9ycyA9IHNwZWNpZXNfY29sb3JzLAogICAgICAgICAgICAgICAgICAgICAgIGNlbGx3aWR0aCA9IDEyLCBjZWxsaGVpZ2h0ID0gMTIsbWFpbiA9IHNwcmludGYoJyVzJywgaSksCiAgICAgICAgICAgICAgICAgICAgICAgIGNvbCA9IGJsdWUyNTUpCn0KYGBgCgojIyMjIEhlYXRtYXAgdXNpbmcgY29ycmVsYXRpb24KCkNsdXN0ZXJpbmcgdXNpbmcgY29ycmVsYXRpb24gYXMgc2ltaWxhcml0eSBtZXRyaWNzIGFyZSBpZGVudGljYWwsIHNpbmNlIGdsb2JhbCBzY2FsaW5nIGRvZXMgbm90IGFmZmVjdCBjb3JyZWxhdGlvbiBiZXR3ZWVuIHNhbXBsZXMuCgpgYGB7cixmaWcuaGVpZ2h0PSA5fQpmb3IgKGkgaW4gbmFtZXMobm9ybS5zdGFuZm9yZCkpIHsKICAgIHggPSBub3JtLnN0YW5mb3JkW1tpXV0kbm9ybWFsaXplZENvdW50cwogICAgY29yKHQoeCksIG1ldGhvZCA9ICdwZWFyc29uJykgJT4lCiAgICAgICAgcGhlYXRtYXA6OnBoZWF0bWFwKGNsdXN0ZXJpbmdfZGlzdGFuY2Vfcm93cyA9ICdjb3JyZWxhdGlvbicsIGNsdXN0ZXJpbmdfZGlzdGFuY2VfY29scyA9ICdjb3JyZWxhdGlvbicsCiAgICAgICAgICAgICAgICAgICAgICAgICAgIGNsdXN0ZXJpbmdfbWV0aG9kID0gJ3NpbmdsZScsCiAgICAgICAgICAgICAgICAgICAgICAgICAgIGFubm90YXRpb25fcm93ID0gZGF0YS5mcmFtZSgnc3BlY2llcycgPSBzdGFuZm9yZC5tZXRhJHNwZWNpZXMsIHJvdy5uYW1lcyA9IHN0YW5mb3JkLm1ldGEkbGFiZWwpLAogICAgICAgICAgICAgICAgICAgICAgICAgICBhbm5vdGF0aW9uX2NvbCA9IGRhdGEuZnJhbWUoJ2JhdGNoJyA9IHN0YW5mb3JkLm1ldGEkc2VxdWVuY2VfaWRlbnRpZmllciwgcm93Lm5hbWVzID0gc3RhbmZvcmQubWV0YSRsYWJlbCksCiAgICAgICAgICAgICAgICAgICAgICAgICAgIGFubm90YXRpb25fY29sb3JzID0gc3BlY2llc19jb2xvcnMsCiAgICAgICAgICAgICAgICAgICAgICAgICAgIGxhYmVsc19yb3cgPSBzdGFuZm9yZC5tZXRhJGxhYmVsLCBsYWJlbHNfY29sID0gc3RhbmZvcmQubWV0YSRzZXF1ZW5jZV9pZGVudGlmaWVyLAogICAgICAgICAgICAgICAgICAgICAgICAgICBjZWxsd2lkdGggPSAxMiwgY2VsbGhlaWdodCA9IDEyLCBtYWluID0gJ05vIGJhdGNoIGNvcnJlY3Rpb24nKQp9CmBgYAoKIyMjIEFmdGVyIGJhdGNoIGNvcnJlY3Rpb24gd2l0aCBjb21iYXQKCiMjIyMgSGVhdG1hcCB1c2luZyBFdWNsaWRlYW4gZGlzdGFuY2UKCmBgYHtyLGZpZy5oZWlnaHQ9OX0KCmZvciAoaSBpbiBuYW1lcyhub3JtLmNvbWJhdC5zdGFuZm9yZCkpIHsKICAgIHggPSBub3JtLmNvbWJhdC5zdGFuZm9yZFtbaV1dCiAgICBkaXN0LnggPSByZW1vdmVfbG93ZXN0X3F1YW50aWxlKHgsIHAgPSAwLjMpICU+JQogICAgICAgIGRpc3QobWV0aG9kID0gJ2V1Y2xpZGVhbicpCiAgICBoY2x1c3QueCA9IGhjbHVzdChkaXN0LngsIG1ldGhvZCA9ICdhdmVyYWdlJykKICAgIHBoZWF0bWFwOjpwaGVhdG1hcChhcy5tYXRyaXgoZGlzdC54KSwKICAgICAgICAgICAgICAgICAgICAgICBjbHVzdGVyX3Jvd3MgPSBoY2x1c3QueCwgY2x1c3Rlcl9jb2xzID0gaGNsdXN0LngsCiAgICAgICAgICAgICAgICAgICAgICAgYW5ub3RhdGlvbl9yb3cgPSBkYXRhLmZyYW1lKCdzcGVjaWVzJyA9IHN0YW5mb3JkLm1ldGEkc3BlY2llcywgcm93Lm5hbWVzID0gc3RhbmZvcmQubWV0YSRsYWJlbCksCiAgICAgICAgICAgICAgICAgICAgICAgYW5ub3RhdGlvbl9jb2wgPSBkYXRhLmZyYW1lKCdiYXRjaCcgPSBzdGFuZm9yZC5tZXRhJHNlcXVlbmNlX2lkZW50aWZpZXIsIHJvdy5uYW1lcyA9IHN0YW5mb3JkLm1ldGEkbGFiZWwpLAogICAgICAgICAgICAgICAgICAgICAgIGFubm90YXRpb25fY29sb3JzID0gc3BlY2llc19jb2xvcnMsCiAgICAgICAgICAgICAgICAgICAgICAgbGFiZWxzX3JvdyA9IHN0YW5mb3JkLm1ldGEkbGFiZWwsIGxhYmVsc19jb2wgPSBzdGFuZm9yZC5tZXRhJHNlcXVlbmNlX2lkZW50aWZpZXIsCiAgICAgICAgICAgICAgICAgICAgICAgY2VsbHdpZHRoID0gMTIsIGNlbGxoZWlnaHQgPSAxMixtYWluID0gc3ByaW50ZignJXMgKyBjb21iYXQnLCBpKSwKICAgICAgICAgICAgICAgICAgICAgICAgY29sID0gYmx1ZTI1NSkKfQpgYGAKCiMjIyMgSGVhdG1hcCB1c2luZyBjb3JyZWxhdGlvbgoKYGBge3IsZmlnLmhlaWdodCA9IDl9CmZvciAoaSBpbiBuYW1lcyhub3JtLmNvbWJhdC5zdGFuZm9yZCkpIHsKICAgIHggPSBub3JtLmNvbWJhdC5zdGFuZm9yZFtbaV1dICU+JQogICAgICAgIHJlbW92ZV9sb3dlc3RfcXVhbnRpbGUocCA9IDAuMykKICAgIGNvcih0KHgpLCBtZXRob2QgPSAncGVhcnNvbicpICU+JQogICAgICAgIHBoZWF0bWFwOjpwaGVhdG1hcChjbHVzdGVyaW5nX2Rpc3RhbmNlX3Jvd3MgPSAnY29ycmVsYXRpb24nLCBjbHVzdGVyaW5nX2Rpc3RhbmNlX2NvbHMgPSAnY29ycmVsYXRpb24nLAogICAgICAgICAgICAgICAgICAgICAgICAgICBjbHVzdGVyaW5nX21ldGhvZCA9ICdzaW5nbGUnLAogICAgICAgICAgICAgICAgICAgICAgICAgICBhbm5vdGF0aW9uX3JvdyA9IGRhdGEuZnJhbWUoJ3NwZWNpZXMnID0gc3RhbmZvcmQubWV0YSRzcGVjaWVzLCByb3cubmFtZXMgPSBzdGFuZm9yZC5tZXRhJGxhYmVsKSwKICAgICAgICAgICAgICAgICAgICAgICAgICAgYW5ub3RhdGlvbl9jb2wgPSBkYXRhLmZyYW1lKCdiYXRjaCcgPSBzdGFuZm9yZC5tZXRhJHNlcXVlbmNlX2lkZW50aWZpZXIsIHJvdy5uYW1lcyA9IHN0YW5mb3JkLm1ldGEkbGFiZWwpLAogICAgICAgICAgICAgICAgICAgICAgICAgICBhbm5vdGF0aW9uX2NvbG9ycyA9IHNwZWNpZXNfY29sb3JzLAogICAgICAgICAgICAgICAgICAgICAgICAgICBsYWJlbHNfcm93ID0gc3RhbmZvcmQubWV0YSRsYWJlbCwgbGFiZWxzX2NvbCA9IHN0YW5mb3JkLm1ldGEkc2VxdWVuY2VfaWRlbnRpZmllciwKICAgICAgICAgICAgICAgICAgICAgICAgICAgY2VsbHdpZHRoID0gMTIsIGNlbGxoZWlnaHQgPSAxMiwgbWFpbiA9IHNwcmludGYoJyVzICsgY29tYmF0JywgaSkpCn0KYGBgCgojIyMjIFZhcnlpbmcgY2x1c3RlcmluZyBhbGdvcml0aG1zIGFuZCBwYXJhbWV0ZXJzCgpOb3JtYWxpemluZyByZWFkIGNvdW50cyBkaWZmZXJlbnRseSBiZWZvcmUgYmF0Y2ggZWZmZWN0IGNvcnJlY3Rpb24gcmVzdWx0ZWQgaW4gZGlmZmVyZW50IGNsdXN0ZXJpbmcgcGF0dGVybnMuIFRvIGNvbXBhcmUgdGhlc2UgcGF0dGVybnMsIHdlIGNob3NlLCByYXRoZXIgYXJiaXRyYXJpbHksIHRoZSBncm91cGluZyB0aGF0IGFsaWducyB3aXRoIHRpc3N1ZSB0eXBlcywgYXMgdGhlIHJlZmVyZW5jZSwgYW5kIHF1YW50aWZ5IHRoZSBzaW1pbGFyaXR5IG9mIGEgY2x1c3RlcmluZyBwYXR0ZXJuIHdpdGggdGhpcyByZWZlcmVuY2UgYnkgYWRqdXN0ZWQgcmFuZCBpbmRleC4gQSBoaWdoZXIgYWRqdXN0ZWQgcmFuZCBpbmRleCBpbmRpY2F0ZXMgbW9yZSBzaW1pbGFyIGNsdXN0ZXJpbmcgcGF0dGVybnMuCgpBc3N1bWluZyB0aGF0IHRoZSBncm91cGluZyBvZiBzYW1wbGVzIGJ5IHRoZWlyIHRpc3N1ZSBvZiBvcmlnaW4gKHJhdGhlciB0aGFuIGJ5IHNwZWNpZXMpIGlzIGEgZGVzaXJhYmxlIGNsdXN0ZXJpbmcgcGF0dGVybiwgYSBoaWdoZXIgYWRqdXN0ZWQgcmFuZCBpbmRleCB0aGVyZWZvcmUgaW5kaWNhdGVzIGEgYmV0dGVyIHByb2Nlc3NlZCBpbnB1dC4gQWx0aG91Z2ggdGhpcyBhc3N1bXB0aW9uIG1heSBub3QgYmUgMTAwJSBhY2N1cmF0ZSBvbiB0aGUgY3VycmVudCBkYXRhIHNldCwgZ2l2ZW4gdGhlIGxvdyBudW1iZXIgb2YgcmVwbGljYXRlcyBhbmQgaGlnaCBsZXZlbCBvZiB2YXJpYXRpb24gaW4gdGhpcyBkYXRhIHNldCBbQENoYW46MjAwOTpDb25zZXJ2YXRpb25dLCBpdCBpcyBndWlkZWQgYnkgdGhlIGNvbnNlcnZhdGlvbiBvZiB0aXNzdWUgdHJhbnNjcmlwdG9taWMgcHJvZmlsZXMgb2JzZXJ2ZWQgYWNyb3NzIG1hbnkgdmVydGVicmF0ZSBzcGVjaWVzLCB1c2luZyBib3RoIG1pY3JvYXJyYXkgW0BDaGFuOjIwMDk6Q29uc2VydmF0aW9uXSBhbmQgUk5BLXNlcSBbQFN1ZG1hbnQ6MjAxNTpNZXRhYW5hbHlzaXMgYW5kIHRoZSBSTkEtc2VxIHN0dWRpZXMgdGhlcmVpbl0uCgpTaW5jZSB0aGUgY2hvaWNlIG9mIGRpc3RhbmNlIG1ldHJpY3MsIGNsdXN0ZXJpbmcgYWxnb3JpdGhtIGFuZCBjbHVzdGVyaW5nIHBhcmFtZXRlcnMgYWxsIGxlYWQgdG8gZGlmZmVyZW50IG91dGNvbWVzLCB3ZSBwZXJmb3JtZWQgY2x1c3RlcmluZyBhdCB2YXJpb3VzIGFsZ29yaXRobXMgYW5kIHBhcmFtZXRlcnMsIGluIG9yZGVyIHRvIHNlZSBpZiB0aGUgZGlmZmVyZW5jZXMgYW1vbmcgdGhvc2UgY2x1c3RlcmluZyBwYXR0ZXJucyByZW1haW4gc3RhYmxlLgpGb3IgSy1tZWFucyBhbmQgc3BlY3RyYWwgY2x1c3RlcmluZyBpbiB3aGljaCB0aGUgY2VudGVycyB3ZXJlIGluaXRpYWxpemVkIHJhbmRvbWx5LCBjbHVzdGVyaW5nIHdhcyByZXBlYXRlZCBtdWx0aXBsZSB0aW1lcyBhbmQgdGhlIGFsbCB0aGUgcmVzdWx0cyB3ZXJlIHJlY29yZGVkIGZvciBjb21wYXJpc29uLgoKRm9yIGVhY2ggY2x1c3RlcmluZyByZXN1bHQsIGFuIGFkanVzdGVkIHJhbmQgaW5kZXggKEFSSSkgd2l0aCByZXNwZWN0IHRvIHJlZmVyZW5jZSAoaS5lLiB0aXNzdWUtYmFzZWQpIGNsdXN0ZXJzIHdhcyBjYWxjdWxhdGVkLCBhbmQgdXNlZCB0byByYW5rIHRoZSBub3JtYWxpemF0aW9uIG1ldGhvZCBhc3NvY2lhdGVkIHdpdGggaXQsIGxvdyBBUkkgY29ycmVzcG9uZHMgdG8gbG93IHJhbmsuIFJhdyBjb3VudHMgKGByYXcgKyBjb21iYXRgKSBjb25zaXN0ZW50bHkgcmFuayBhdCB0aGUgYm90dG9tLCBwcmFjdGljYWxseSBub3QgYWZmZWN0ZWQgYnkgYWxnb3JpdGhtaWMgYW5kIHBhcmFtZXRyaWMgY2hvaWNlcyBvZiBjbHVzdGVyaW5nLiBUaGUgZGlmZmVyZW5jZXMgaW4gcmFua2luZyBiZXR3ZWVuIG5vcm1hbGl6YXRpb24gbWV0aG9kcyBhcmUgbGVzcyBjbGVhciBhbmQgbGVzcyBjb25zaXN0ZW50LgoKIyMjIyMgSGllcmFyY2hpY2FsIGNsdXN0ZXJpbmcKCmBgYHtyfQpjbHVzdC5ub3JtLmNvbWJhdCA9IGxpc3QoKQpgYGAKCmBgYHtyfQpobCA9IGxhcHBseShuYW1lcyhub3JtLmNvbWJhdC5zdGFuZm9yZCksIGZ1bmN0aW9uKGkpIHsKICAgIHggPSBub3JtLmNvbWJhdC5zdGFuZm9yZFtbaV1dCiAgICByZW1vdmVfbG93ZXN0X3F1YW50aWxlKHgpICU+JQogICAgICAgIGRpc3QobWV0aG9kID0gJ2V1Y2xpZGVhbicpICU+JQogICAgICAgIGhjbHVzdCgpCn0pICU+JQogICAgc2V0X25hbWVzKG5hbWVzKG5vcm0uY29tYmF0LnN0YW5mb3JkKSkgJT4lCiAgICBzYXBwbHkoZnVuY3Rpb24oeCkgewogICAgY3V0cmVlKHgsIGsgPSAxMykgJT4lCiAgICBtY2x1c3Q6OmFkanVzdGVkUmFuZEluZGV4KHN0YW5mb3JkLm1ldGEkdGlzc3VlKQp9KSAlPiUKICAgIHNldF9uYW1lcyhuYW1lcyhub3JtLmNvbWJhdC5zdGFuZm9yZCkpICU+JQogICAgbGlzdCgncmVzdWx0JyA9IC4sCiAgICAgICAgICdhbGdvJyA9ICdIaWVyYXJjaGljYWwgY2x1c3RlcmluZycsCiAgICAgICAgICdSIGZ1bmN0aW9uJyA9ICdoY2x1c3QnLAogICAgICAgICAncGFyYW1ldGVycycgPSAnbWV0aG9kPWNvbXBsZXRlO2Rpc3Q9ZXVjbGlkZWFuJykKY2x1c3Qubm9ybS5jb21iYXQgPSBhcHBlbmQoY2x1c3Qubm9ybS5jb21iYXQsIGxpc3QoaGwpKQpobCRyZXN1bHQKYGBgCgpgYGB7cn0KaGwgPSBsYXBwbHkobmFtZXMobm9ybS5jb21iYXQuc3RhbmZvcmQpLCBmdW5jdGlvbihpKSB7CiAgICB4ID0gbm9ybS5jb21iYXQuc3RhbmZvcmRbW2ldXQogICAgcmVtb3ZlX2xvd2VzdF9xdWFudGlsZSh4KSAlPiUKICAgICAgICBkaXN0KG1ldGhvZCA9ICdldWNsaWRlYW4nKSAlPiUKICAgICAgICBoY2x1c3QobWV0aG9kID0gJ2F2ZXJhZ2UnKQp9KSAlPiUKICAgIHNldF9uYW1lcyhuYW1lcyhub3JtLmNvbWJhdC5zdGFuZm9yZCkpICU+JQogICAgc2FwcGx5KGZ1bmN0aW9uKHgpIHsKICAgIGN1dHJlZSh4LCBrID0gMTMpICU+JQogICAgbWNsdXN0OjphZGp1c3RlZFJhbmRJbmRleChzdGFuZm9yZC5tZXRhJHRpc3N1ZSkKfSkgJT4lCiAgICBzZXRfbmFtZXMobmFtZXMobm9ybS5jb21iYXQuc3RhbmZvcmQpKSAlPiUKICAgIGxpc3QoJ3Jlc3VsdCcgPSAuLAogICAgICAgICAnYWxnbycgPSAnSGllcmFyY2hpY2FsIGNsdXN0ZXJpbmcnLAogICAgICAgICAnUiBmdW5jdGlvbicgPSAnaGNsdXN0JywKICAgICAgICAgJ3BhcmFtZXRlcnMnID0gJ21ldGhvZD1hdmVyYWdlO2Rpc3Q9ZXVjbGlkZWFuJykKY2x1c3Qubm9ybS5jb21iYXQgPSBhcHBlbmQoY2x1c3Qubm9ybS5jb21iYXQsIGxpc3QoaGwpKQpgYGAKCmBgYHtyfQpobCA9IGxhcHBseShuYW1lcyhub3JtLmNvbWJhdC5zdGFuZm9yZCksIGZ1bmN0aW9uKGkpIHsKICAgIHggPSBub3JtLmNvbWJhdC5zdGFuZm9yZFtbaV1dCiAgICByZW1vdmVfbG93ZXN0X3F1YW50aWxlKHgpICU+JQogICAgICAgIGRpc3QobWV0aG9kID0gJ21hbmhhdHRhbicpICU+JQogICAgICAgIGhjbHVzdChtZXRob2QgPSAnY29tcGxldGUnKQp9KSAlPiUKICAgIHNldF9uYW1lcyhuYW1lcyhub3JtLmNvbWJhdC5zdGFuZm9yZCkpICU+JQogICAgc2FwcGx5KGZ1bmN0aW9uKHgpIHsKICAgIGN1dHJlZSh4LCBrID0gMTMpICU+JQogICAgbWNsdXN0OjphZGp1c3RlZFJhbmRJbmRleChzdGFuZm9yZC5tZXRhJHRpc3N1ZSkKfSkgJT4lCiAgICBzZXRfbmFtZXMobmFtZXMobm9ybS5jb21iYXQuc3RhbmZvcmQpKSAgJT4lCiAgICBsaXN0KCdyZXN1bHQnID0gLiwKICAgICAgICAgJ2FsZ28nID0gJ0hpZXJhcmNoaWNhbCBjbHVzdGVyaW5nJywKICAgICAgICAgJ1IgZnVuY3Rpb24nID0gJ2hjbHVzdCcsCiAgICAgICAgICdwYXJhbWV0ZXJzJyA9ICdtZXRob2Q9Y29tcGxldGU7ZGlzdD1tYW5oYW50dGFuJykKY2x1c3Qubm9ybS5jb21iYXQgPSBhcHBlbmQoY2x1c3Qubm9ybS5jb21iYXQsIGxpc3QoaGwpKQpgYGAKYGBge3J9CmhsID0gbGFwcGx5KG5hbWVzKG5vcm0uY29tYmF0LnN0YW5mb3JkKSwgZnVuY3Rpb24oaSkgewogICAgeCA9IG5vcm0uY29tYmF0LnN0YW5mb3JkW1tpXV0KICAgIHJlbW92ZV9sb3dlc3RfcXVhbnRpbGUoeCkgJT4lCiAgICAgICAgZGlzdChtZXRob2QgPSAnbWFuaGF0dGFuJykgJT4lCiAgICAgICAgaGNsdXN0KG1ldGhvZCA9ICdhdmVyYWdlJykKfSkgJT4lCiAgICBzZXRfbmFtZXMobmFtZXMobm9ybS5jb21iYXQuc3RhbmZvcmQpKSAlPiUKICAgIHNhcHBseShmdW5jdGlvbih4KSB7CiAgICBjdXRyZWUoeCwgayA9IDEzKSAlPiUKICAgIG1jbHVzdDo6YWRqdXN0ZWRSYW5kSW5kZXgoc3RhbmZvcmQubWV0YSR0aXNzdWUpCn0pICU+JQogICAgc2V0X25hbWVzKG5hbWVzKG5vcm0uY29tYmF0LnN0YW5mb3JkKSkgICU+JQogICAgbGlzdCgncmVzdWx0JyA9IC4sCiAgICAgICAgICdhbGdvJyA9ICdIaWVyYXJjaGljYWwgY2x1c3RlcmluZycsCiAgICAgICAgICdSIGZ1bmN0aW9uJyA9ICdoY2x1c3QnLAogICAgICAgICAncGFyYW1ldGVycycgPSAnbWV0aG9kPWF2ZXJhZ2U7ZGlzdD1tYW5oYW50dGFuJykKY2x1c3Qubm9ybS5jb21iYXQgPSBhcHBlbmQoY2x1c3Qubm9ybS5jb21iYXQsIGxpc3QoaGwpKQpobCRyZXN1bHQKYGBgCgpgYGB7cn0KaGwgPSBsYXBwbHkobmFtZXMobm9ybS5jb21iYXQuc3RhbmZvcmQpLCBmdW5jdGlvbihpKSB7CiAgICB4ID0gbm9ybS5jb21iYXQuc3RhbmZvcmRbW2ldXSAlPiUKICAgICAgICByZW1vdmVfbG93ZXN0X3F1YW50aWxlKHAgPSAwLjMpCiAgICAoMSAtIGNvcih0KHgpKSkgJT4lCiAgICAgICAgYXMuZGlzdCgpICU+JQogICAgICAgIGhjbHVzdChtZXRob2QgPSAnc2luZ2xlJykKfSkgJT4lCiAgICBzZXRfbmFtZXMobmFtZXMobm9ybS5jb21iYXQuc3RhbmZvcmQpKSAlPiUKICAgIHNhcHBseShmdW5jdGlvbih4KSB7CiAgICBjdXRyZWUoeCwgayA9IDEzKSAlPiUKICAgIG1jbHVzdDo6YWRqdXN0ZWRSYW5kSW5kZXgoc3RhbmZvcmQubWV0YSR0aXNzdWUpCn0pICU+JQogICAgc2V0X25hbWVzKG5hbWVzKG5vcm0uY29tYmF0LnN0YW5mb3JkKSkgJT4lCiAgICBsaXN0KCdyZXN1bHQnID0gLiwKICAgICAgICAgJ2FsZ28nID0gJ0hpZXJhcmNoaWNhbCBjbHVzdGVyaW5nJywKICAgICAgICAgJ1IgZnVuY3Rpb24nID0gJ2hjbHVzdCcsCiAgICAgICAgICdwYXJhbWV0ZXJzJyA9ICdtZXRob2Q9c2luZ2xlO2Rpc3Q9MS1jb3InKQpjbHVzdC5ub3JtLmNvbWJhdCA9IGFwcGVuZChjbHVzdC5ub3JtLmNvbWJhdCwgbGlzdChobCkpCmhsJHJlc3VsdApgYGAKCgojIyMjIyBLLW1lYW5zCgpgYGB7cn0KTl9SRVBFQVRTID0gMjAKY2wgPSBsYXBwbHkobmFtZXMobm9ybS5jb21iYXQuc3RhbmZvcmQpLCBmdW5jdGlvbihpKSB7CiAgICB4ID0gbm9ybS5jb21iYXQuc3RhbmZvcmRbW2ldXSAlPiUKICAgICAgICByZW1vdmVfbG93ZXN0X3F1YW50aWxlKHAgPSAwLjMpCiAgICBsYXBwbHkoMTpOX1JFUEVBVFMsIGZ1bmN0aW9uKGopIHsKICAgICAgICBrbCA9IGttZWFucyh4LCBjZW50ZXJzID0gMTMsIGl0ZXIubWF4ID0gNTApCiAgICAgICAgcmV0dXJuKGtsJGNsdXN0ZXIpCiAgICB9KQp9KSAlPiUKICAgIHNldF9uYW1lcyhuYW1lcyhub3JtLmNvbWJhdC5zdGFuZm9yZCkpICU+JQogICAgbGFwcGx5KGZ1bmN0aW9uKHgpIHsKICAgICAgICBzYXBwbHkoeCwgZnVuY3Rpb24oeF9qKSB7CiAgICAgICAgICAgIG1jbHVzdDo6YWRqdXN0ZWRSYW5kSW5kZXgoeF9qLCBzdGFuZm9yZC5tZXRhJHRpc3N1ZSkKICAgICAgICB9KQogICAgfSkgJT4lCiAgICBzZXRfbmFtZXMobmFtZXMobm9ybS5jb21iYXQuc3RhbmZvcmQpKSAgJT4lCiAgICBkby5jYWxsKHJiaW5kLCAuKSAlPiUKICAgIGxpc3QoJ3Jlc3VsdCcgPSAuLAogICAgICAgICAnYWxnbycgPSAnSy1tZWFucycsCiAgICAgICAgICdSIGZ1bmN0aW9uJyA9ICdzdGF0czo6a21lYW5zJywKICAgICAgICAgJ3BhcmFtZXRlcnMnID0gJ21ldGhvZD1IYXRpZ2FuLVdvbmc7IGsgPSAxMycpCmNsdXN0Lm5vcm0uY29tYmF0ID0gYXBwZW5kKGNsdXN0Lm5vcm0uY29tYmF0LCBsaXN0KGNsKSkKY2wkcmVzdWx0CmBgYAoKIyMjIyMgU3BlY3RyYWwgY2x1c3RlcmluZwoKYGBge3J9Ck5fUkVQRUFUUyA9IDIwCnNjID0gbGFwcGx5KG5hbWVzKG5vcm0uY29tYmF0LnN0YW5mb3JkKSwgZnVuY3Rpb24oaSkgewogICAgeCA9IG5vcm0uY29tYmF0LnN0YW5mb3JkW1tpXV0gJT4lCiAgICAgICAgcmVtb3ZlX2xvd2VzdF9xdWFudGlsZShwID0gMC4zKQogICAgbGFwcGx5KDE6Tl9SRVBFQVRTLCBmdW5jdGlvbihqKSB7CiAgICAgICAgcmV0dXJuKGtlcm5sYWI6OnNwZWNjKHgsIGNlbnRlcnMgPSAxMykpCiAgICB9KQp9KSAlPiUgCiAgICBzZXRfbmFtZXMobmFtZXMobm9ybS5jb21iYXQuc3RhbmZvcmQpKSAlPiUKICAgIGxhcHBseShmdW5jdGlvbih4KSB7CiAgICAgICAgc2FwcGx5KHgsIGZ1bmN0aW9uKHhfaikgewogICAgICAgICAgICBhcy5udW1lcmljKHhfaikgJT4lCiAgICAgICAgICAgICAgICBtY2x1c3Q6OmFkanVzdGVkUmFuZEluZGV4KHN0YW5mb3JkLm1ldGEkdGlzc3VlKQogICAgICAgIH0pICAgICAgICAKICAgIH0pICU+JQogICAgc2V0X25hbWVzKG5hbWVzKG5vcm0uY29tYmF0LnN0YW5mb3JkKSkgICU+JQogICAgZG8uY2FsbChyYmluZCwgLikgJT4lCiAgICBsaXN0KCdyZXN1bHQnID0gLiwKICAgICAgICAgJ2FsZ28nID0gJ1NwZWN0cmFsIGNsdXN0ZXJpbmcnLAogICAgICAgICAnUiBmdW5jdGlvbicgPSAna2VybmxhYjo6c3BlY3RyYWwnLAogICAgICAgICAncGFyYW1ldGVycycgPSAnaz0xMycpCmNsdXN0Lm5vcm0uY29tYmF0ID0gYXBwZW5kKGNsdXN0Lm5vcm0uY29tYmF0LCBsaXN0KHNjKSkKYGBgCgojIyMjIFNpbWlsYXJpdHkgd2l0aCB0aXNzdWUtYmFzZWQgY2x1c3RlcnMKClNpbWlsYXJpdHkgd2l0aCB0aGUgdGlzc3VlLWJhc2VkIGNsdXN0ZXJzIGlzIG1lYXN1cmVkIGJ5IGFkanVzdGVkIHJhbmQgaW5kZXguCgpgYGB7cn0KY2xfc3VtbWFyeS5kZiA9IGxhcHBseShjbHVzdC5ub3JtLmNvbWJhdCwgZnVuY3Rpb24oeCkgewogICAgc3RyKHgpCiAgICBkYXRhLmZyYW1lKCdBbGdvcml0aG0nID0geCRhbGdvLAogICAgICAgICAgICAgICAnUiBmdW5jdGlvbicgPSB4JGBSIGZ1bmN0aW9uYCwKICAgICAgICAgICAgICAgJ0NsdXN0ZXJpbmcgcGFyYW1ldGVycycgPSB4JHBhcmFtZXRlcnMsIGNoZWNrLm5hbWVzID0gRkFMU0UpICU+JQogICAgICAgIGNiaW5kKGRhdGEuZnJhbWUodCh4JHJlc3VsdCkpKQp9KSAlPiUKICAgIGRvLmNhbGwocmJpbmQsIC4pCmNsX3N1bW1hcnkuZGYKYGBgCgpSYW5rIGNvcnJlbGF0aW9uIGJldHdlZW4gY2x1c3RlcmluZyBtZXRob2RzCgpgYGB7cixmaWcuaGVpZ2h0PTEwfQpjb3IucmFuayA9IGNsX3N1bW1hcnkuZGZbLG5hbWVzKG5vcm0uY29tYmF0LnN0YW5mb3JkKV0gJT4lCiAgICB0KCkgJT4lCiAgICBjb3IobWV0aG9kID0gJ3NwZWFybWFuJykKcGhlYXRtYXA6OnBoZWF0bWFwKDEgLSBjb3IucmFuaywKICAgICAgICAgICAgICAgICAgIGNlbGx3aWR0aCA9IDEwLAogICAgICAgICAgICAgICAgICAgY2VsbGhlaWdodCA9IDEwLAogICAgICAgICAgICAgICAgICAgbGFiZWxzX3JvdyA9IGNsX3N1bW1hcnkuZGYkQWxnb3JpdGhtLAogICAgICAgICAgICAgICAgICAgbGFiZWxzX2NvbCA9IGNsX3N1bW1hcnkuZGYkQWxnb3JpdGhtKQpgYGAKCkRpZmZlcmVudGx5IG5vcm1hbGl6ZWQgY291bnRzIHJlc3VsdGVkIGluIGRpZmZlcmVudCBjbHVzdGVyaW5nIHBhdHRlcm5zLiBFYWNoIGNvbmZpZ3VyYXRpb24gb2Ygbm9ybWFsaXphdGlvbiBtZXRob2RzCgpgYGB7cn0KY2xfc3VtbWFyeS5kZlssbmFtZXMobm9ybS5jb21iYXQuc3RhbmZvcmQpXSAlPiUKICAgIGFwcGx5KE1BUkdJTiA9IDEsIEZVTiA9IHJhbmspICU+JQogICAgdCgpICU+JQogICAgc3VtbWFyeSgpCmBgYAoKCgpgYGB7cn0Kc2Vzc2lvbkluZm8oKQpgYGAKCiMjIFJlZmVyZW5jZXM=
