# Supplementary material for: A graph-based algorithm for RNA-seq data normalization
Source: PLoS One. 2020 Jan 24;15(1):e0227760. doi: 10.1371/journal.pone.0227760 (PMC6980396; doi:10.1371/journal.pone.0227760)
Supplement: Attachment — Submitted filename: Response to reviewers.pdf [file pone.0227760.s004.pdf]

**Reviewer #1:**

I believe that this is a novel and interesting approach to gene expression normalization. While I don't think there is anything theoretically incorrect with your implementation, I believe there should be one more comparative method and at least one additional metric (independent of spike-in values) with which you score your model to ensure it works in practice.

Here are my two main concerns with the manuscript:

1) Dependence on spike-in values for evaluation. Your method does not adequately show that your algorithm addresses the problem that normalization attempts to answer - to remove unwanted variation - i.e. variation that is irrelevant to the experiment performed. In this paper you use spike-ins as the gold standard, however, it has been reported many times spike-ins have a high variability within replicates (Qing. et. al 2013 "mRNA enrichment protocols determine the quantification characteristics of external RNA spike-in controls in RNA-Seq studies."). I believe that your method may still be useful, but the metrics you use are only related to reconstructing spike-in expression, or identifying the spike-ins as controls. This could potentially be very misleading, since reconstructing the spike-in values could potentially be biased. It would be much more convincing if you could include another metric that is independent of the spike-ins/control genes, but shows that the downstream analyses are improved using your method vs. other methods. One such example would be to show that your method removes technical variation, such as batch effects. This has been nicely done on ENCODE expression data, which you already have experience with, by showing that the gene expression of each tissue clusters with one another instead of by species (mouse vs. human). This has been discussed in (Gilad et. al. 2015 "A reanalysis of mouse ENCODE comparative gene expression data"). If your method was to outperform their normalization, in terms of clustering tissues more closely together instead of species, this would be more convincing. Two metrics commonly used to judge clusters are the adjusted rand index or silhouette score.

We have added analysis on the batch-confounded data set of human and mouse tissues, showing that batch correction is improved after normalization (compared to batch correction performed directly on un-normalized counts), in the sense that samples are clustered by tissues rather than by species. This facilitating role is observed with all other global scaling normalization methods that we included in the extended analysis (Supplementary R notebook).

It should be noted that although spike-ins are used to establish the ground truth, this ground truth is only defined in the context of global scaling normalization. If technical biases affect genes in a

non-linear fashion, a single scaling factor per sample won't be able to correct for such effect. Hence it is reasonable to combine global scaling normalization with a batch effect correction algorithm in an RNA-seq processing pipeline, instead of considering them as processing alternatives.

We recognize that spike-ins can be used in a number of ways, some of which may lead to the expectation that spike-ins help eliminate all unwanted variations, hence correcting for batch effects. For that reason, we have extended the Discussion section to clarify these various uses, as well as how the procedure described in the manuscript fits in a broader context of RNA-seq processing pipelines.

2) There currently exists a method very similar to your proposed method called RUVSeq (Risso et. al. 2015 "Normalization of RNA-seq data using factor analysis of control genes or samples"), which also uses control genes (spike-ins or computationally identified) to normalize the data. However it differs in the implementation: Risso et. al. uses a linear modeling approach. Since this method is very similar and it is used in the field (531 citations), I believe it is the most relevant method to compare against. It may also be useful to evaluate your method in a similar way that RUVSeq does.

RUV seems similar to gbnorm in their reliance on a reference set (RUVg variant). However, in contrast to all the methods examined in this work (including gbnorm), RUV allows for both sample- and gene-specific scaling factors. This working leads to a highly different expression matrix which is incomparable using *cdev*. For a perspective, the deviation of RUVg-normalized counts is on the order of 10^2 while all other global-scaling methods resulted in *cdev* on the order of 10. As shown in the supplementary R notebook, cdev is only a reliable indicator of noise variance when it's below 100. As this does not necessarily mean that RUVg will perform worse in downstream analysis, we think it would be misleading to include RUV in the comparison.

On the other hand, RUVg may use an empirical set of references which can be determined using other methods. Such usage brings it closer to the realm of batch effect correction than global scaling normalization.

We recognize that the term "normalization" encircles a very broad range of transformations, from global scaling to factor analysis and quantile normalization, and such common term sometimes obscures the differences. For clarification, the Discussion section is now extended to consider global scaling normalization in the context of a broad sense "normalization".

Minor comments:

- You have a section called "Preliminary results", why are these preliminary?

The section is now “Results”.

- Can you share the code for gbnorm? The details are available, but having the code will make it much more widely used, perhaps putting it on Bioconductor would be useful or maybe an R notebook.

The code of gbnorm will be made public on github (or public repository available at the time) should the manuscript be accepted.

- A very good use case for this data would be in cancer studies, where the assumption that a majority of the genes are not differential can be violated. It may be useful to do one differential expression analysis on TCGA count data on a large dataset that has a known artifact (sampled by two labs, or a later release of the TCGA data)

Although RNA-seq data from TCGA have been made available and even re-processed by different groups, we are not aware of a data source where the metadata is provided with sufficient details to trace back and remove batch effects. We have, instead, included the analysis on the ENCODE data set of 26 samples of human-mouse tissues on which the batch has been annotated and the downstream effects can be recognized via the clustering pattern of samples.

- In Table 2 the result doesn't seem very convincing given the drastic increase in runtime. Maybe this is because I don't really have an intuitive sense of the magnitude of cdev. If you could include an illustration of how a difference of ~1 cdev is significant for downstream analyses this can also improve the impact of the paper.

We are aware that the running time is significantly longer than the existing methods, hence do not recommend running graph-based normalization for every use case. Also, when the clustering algorithm did not converge (as in the case of *gbnorm* applied on the Full Spiked set), the normalization result was not as good as that of earlier methods. Graph partitioning (i.e. clustering) is a difficult problem on its own. As we noted in the manuscript, the specific choice of graph partitioning method and the parameters associated with the chosen method can lead to varying performance. We anticipate this exploration to be a major effort and are planning to pursue it in follow-up work.

To help interpreting *cdev*, we now provide a Supplementary R Notebook exploring how *cdev* varies with the amount of noise added to the Identity matrix, assuming either normally distributed or uniformly distributed noise: log(*cdev*) is linearly proportional to the variance of the noise added to an identity matrix, up to 100. Beyond that, the differences are too large and *cdev* is no longer a good measure of differences.

### Reviewer #2

1. I disagree with the authors' statement that 'inclusion of differential feature in normalizing factors leas to invalid normalization'. Normalization does not remove biological variation or phenotype effects if these factors or conditions are separated from technical artifacts by a valid statistical model. For example, Bioconductor package sva detects latent technical batch effect on RNA-seq counts in sva() and removes the noise via ComBat().

Although “normalization” in the gene expression profiling literature may indicate very different operations on the count matrix, we restrict the meaning of “normalization” to the operation that scales the matrix by sample-specific scaling factors. Hence, in the context of this paper and along the lines of given definitions, the statement is to be taken with a narrower sense, i.e. “inclusion of differential features in the *global scaling* factors leads to invalid normalization”.

We recognize that the term “normalization” encircles a very broad range of transformations, from global scaling to factor analysis and quantile normalization, and such common term sometimes obscures their differences. For clarification, the Discussion section is now extended to consider global scaling normalization in the context of a broad sense “normalization”.

2. The author suggested to use a subset of references for normalization. What does a subset reference mean in this paper? Is it mRNA only, non-coding RNA only, spike-in RNA only?

For the analysis presented in this paper, we defined **references** as the genes/transcripts that *are expressed at equivalent levels across conditions*. As explained in the manuscript,

> *From the biological standpoint, reference genes/transcripts should be constitutive, or at the least, are not under the biological regulations that distinguish the conditions. It should be noted that, for the purpose of normalizing read counts, reference genes/transcripts are numerically stable across conditions, and are not necessarily related to housekeeping genes/transcripts.*

By this definition, the references used in this manuscript are equivalent to the term “negative controls” in the context of differential expression analysis.

Hence the “subset of references” effectively means any number of genes/transcripts that are numerically stable across conditions, regardless of the RNA type or its origin.

3. I suggest the authors to reformat the manuscript as introduction, methods, results, discussion, conclusion. The current format is difficult to read. The statements in Theorem, Observations should be merged with other paragraphs. I don't see the necessity to present the 'proofs' in the main text. Besides, these statements are not exactly mathematical theorems and the equations (1)-(6) should not be labeled as 'proof'.

The manuscript is now re-structured, with a dedicated section for the formulation of graph-based normalization, plus Methods, Results, and an extended Discussion. As we feel strongly about making explicit all the assumptions and modeling decisions made in the reasoning process, we decided to retain most of the “Formulation” section in the main text.
