# Supplementary material for: A graph-based algorithm for RNA-seq data normalization
Source: PLoS One. 2020 Jan 24;15(1):e0227760. doi: 10.1371/journal.pone.0227760 (PMC6980396; doi:10.1371/journal.pone.0227760)
Supplement: Attachment — Submitted filename: Response to reviewers.pdf [file pone.0227760.s005.pdf]

Reviewer #2: The authors have revised the manuscript thoroughly and the methods and algorithms are now clearly described. However, there are major errors or concerns in the proof for Theorem 1 and the selection of performance metrics, being a red flag for the validity of this algorithm.

1. Equations (1) and (2) are NOT equivalent, because c_ij /M_i is NOT proportional (or equal) to a^rel_ij. c_ij is the read counts computed by RNA-Seq bioinformatics software, which aligns and maps the reads to genome reference and may result in random discrepancy in aligning and mapping. Hence, c_ij is not a value by multiplying a_ij with a constant not related to i or j. The author did not assume c_ij as a variable randomly deviating from the true abundance a_ij, which was problematic and lack of statistical validity. This also indicates that the statement 'a*_ij=constant * a_ij' may not be valid.

It was not clear to us what was meant by "random discrepancy in aligning and mapping" caused by the alignment and mapping software. However, quantification (read mapping and counting) is an upstream processing step that has been treated separately from normalization. Although different choices of quantification method can lead to different results, it is reasonable to assume that by the time read counts were obtained, sequencing bias (e.g. positional bias, sequence compositional bias, etc.) have been modeled. The model in RSEM provides an instructive example in what biases are typically modeled in the quantification step (Li & Dewey, 2011).

After quantification, the "main reason for normalization", as put forth by Robinson & Oshlack (2010), is the "true biological differences in RNA composition between samples", *RNA composition* here is equivalent to our term *relative abundance*. Following these authors, and in our annotations, read counts can be modeled as a function of feature abundance, feature length, and sequencing depth,

$$c_{ij} = \frac{a_{ij}\ell_j M_i}{\sum_{g\in all} a_{ig}\ell_g}$$

When the part $\frac{a_{ij}\ell_j}{\sum_{g\in all} a_{ig}\ell_g}$ is wrapped in a quantity called length-adjusted relative abundance, the model reduces to the one we are using in the manuscript, with an identical proof for Theorem 1. We have added a paragraph to elaborate on this connection.

Regarding the concern that c_ij was "not assumed [to be a random variable]", we consider it a modeling decision. For example, in the model by Robinson & Oshlack, read count is described as a random variable depending on feature abundance, feature length, and sequencing depth. More generally, read count X follows a distribution with unknown parameters $\theta_1, \theta_2, \ldots, \theta_n$. One may go further to treat $\theta's$as random variables, or assume $\theta's$are constants. These choices represent two different views in statistical modeling, namely the Bayesian and the frequentist view. Since the debate over which one to take can be philosophical in nature, we rather take a practical standpoint here, by asking whether a model is helpful for its purpose. We chose to treat feature abundances (and read counts) as condition-specific constants, and found it sufficient to predict the artificial correlations among reference features (Figure 1 in the manuscript). Despite its simplicity, this treatment is compatible with the random-variable model, by replacing the constants (a_ij and c_ij) by their means. To improve clarity, we have re-arranged some texts. In the section *Normalization by references*, we now add this statement:

> *For simplicity, feature abundance is treated as condition-specific constant, that is, assuming biological and technical variance across replicates of the same condition is zero. The operations remain the same when abundance levels and*

read counts are treated as random variables, by replacing the relevant constants by the expectations of their random-variable counterparts.

We were aware of this simplification, and thus have asserted in the Discussion section:

Using a simplistic treatment of RNA-seq read counts, we showed that artificial correlations are theoretically expected, and experimentally observed in RNA-seq data.

2. The performance metrics did not measure the removal of batch effect, due to the incorrect assumption a*_ij=constant * a_ij. The best normalization performance metric is coefficient of variation (CV) for normalized counts of reference features.

As explained earlier, the goal of between-sample normalization is to recover the pattern of differential (and non-differential) expression, but not to remove batch effects. On the other hand, due to the complicated influence of batch effects, most judgments about batch effects were made in a qualitative way, for example by investigating PCA/MDS plot to see if the major sources of variances still align with any batch, or by checking if the analytic outcome contradicts with previous knowledge (e.g. clustering of tissue samples by species). That said, the removal of batch effects is beyond the scope of this paper.

We disagree with the statement that the "*best normalization performance metric is coefficient of variation (CV) for normalized counts of reference features*". Since normalization procedures eventually reduce the variation of the reference features, their CV will certainly decrease. As illustrated in Figure 1 below, CV of reference features always reduce, regardless of how many differential genes are mistakenly included in the reference set. In other words, CV of reference features is more of an "objective function" being minimized by the normalization procedure, and is not fit to be a measure of performance.

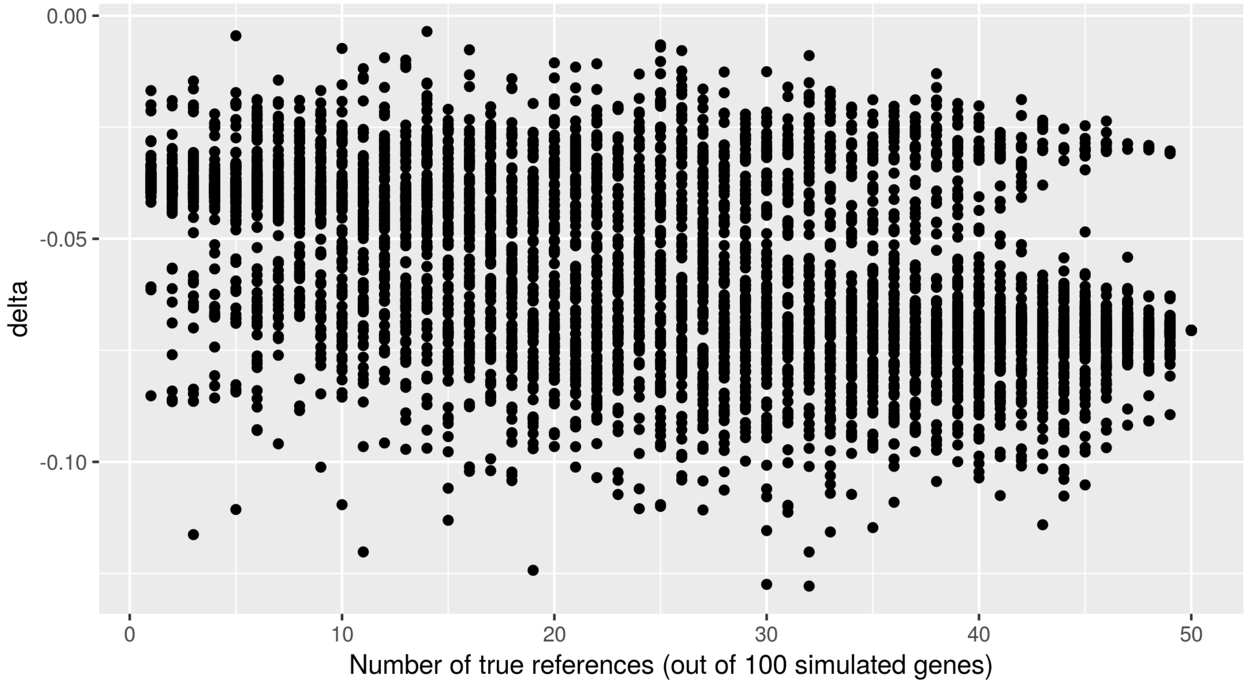

Figure 1. The change in CV of reference genes (delta = CV_after - CV_before), after read counts are normalized by a set of 50 references, only x of which are true references. Simulated reads were generated for 20 samples x 100 genes, 50% of which are set to be non-differential (i.e. references). Simulation code was provided at https://github.com/ciaranlevans/rnaSeqAssumptions as part of the publication by Evans, Hardin, & Stoebel (2017), cited in the manuscript.

References

Evans, C., Hardin, J., & Stoebel, D. M. (2017). Selecting between-sample RNA-Seq normalization methods from the perspective of their assumptions. *Briefings in Bioinformatics*. https://doi.org/10.1093/bib/bbx008

Li, B., & Dewey, C. N. (2011). RSEM: Accurate transcript quantification from RNA-Seq data with or without a reference genome. *BMC Bioinformatics*, *12*, 323. https://doi.org/10.1186/1471-2105-12-323

Robinson, M. D., & Oshlack, A. (2010). A scaling normalization method for differential expression analysis of RNA-seq data. *Genome Biology*, *11*, R25. https://doi.org/10.1186/gb-2010-11-3-r25
